# The Crystal Structure and Small-Angle X-Ray Analysis of CsdL/TcdA Reveal a New tRNA Binding Motif in the MoeB/E1 Superfamily

**DOI:** 10.1371/journal.pone.0118606

**Published:** 2015-04-21

**Authors:** Miguel López-Estepa, Ana Ardá, Martin Savko, Adam Round, William E. Shepard, Marta Bruix, Miquel Coll, Francisco J. Fernández, Jesús Jiménez-Barbero, M. Cristina Vega

**Affiliations:** 1 Chemical and Physical Biology, Center for Biological Research (CIB-CSIC), Madrid, Spain; 2 Structural Biology, CIC bioGUNE, Derio-Bizkaia, Spain; 3 Synchrotron SOLEIL, Gif-sur-Yvette, France; 4 European Molecular Biology Laboratory, Grenoble Outstation, Grenoble, France; 5 Unit for Virus Host-Cell Interactions, Univ. Grenoble Alpes-EMBL-CNRS, Martyrs, France; 6 Institute of Physical Chemistry “Rocasolano” (IFQR-CSIC), Madrid, Spain; 7 Institute for Research in Biomedicine (IRB Barcelona), Barcelona, Spain; 8 Institut de Biologia Molecular de Barcelona (IBMB-CSIC), Barcelona, Spain; Universitetet i Bergen, NORWAY

## Abstract

Cyclic *N* ^6^-threonylcarbamoyladenosine (‘cyclic t^6^A’, ct^6^A) is a non-thiolated hypermodification found in transfer RNAs (tRNAs) in bacteria, protists, fungi and plants. In bacteria and yeast cells ct^6^A has been shown to enhance translation fidelity and efficiency of ANN codons by improving the faithful discrimination of aminoacylated tRNAs by the ribosome. To further the understanding of ct^6^A biology we have determined the high-resolution crystal structures of CsdL/TcdA in complex with AMP and ATP, an E1-like activating enzyme from *Escherichia coli*, which catalyzes the ATP-dependent dehydration of t^6^A to form ct^6^A. CsdL/TcdA is a dimer whose structural integrity and dimer interface depend critically on strongly bound K^+^ and Na^+^ cations. By using biochemical assays and small-angle X-ray scattering we show that CsdL/TcdA can associate with tRNA with a 1:1 stoichiometry and with the proper position and orientation for the cyclization of t^6^A. Furthermore, we show by nuclear magnetic resonance that CsdL/TcdA engages in transient interactions with CsdA and CsdE, which, in the latter case, involve catalytically important residues. These short-lived interactions may underpin the precise channeling of sulfur atoms from cysteine to CsdL/TcdA as previously characterized. In summary, the combination of structural, biophysical and biochemical methods applied to CsdL/TcdA has afforded a more thorough understanding of how the structure of this E1-like enzyme has been fine tuned to accomplish ct^6^A synthesis on tRNAs while providing support for the notion that CsdA and CsdE are able to functionally interact with CsdL/TcdA.

## Introduction

**Fig 1:**
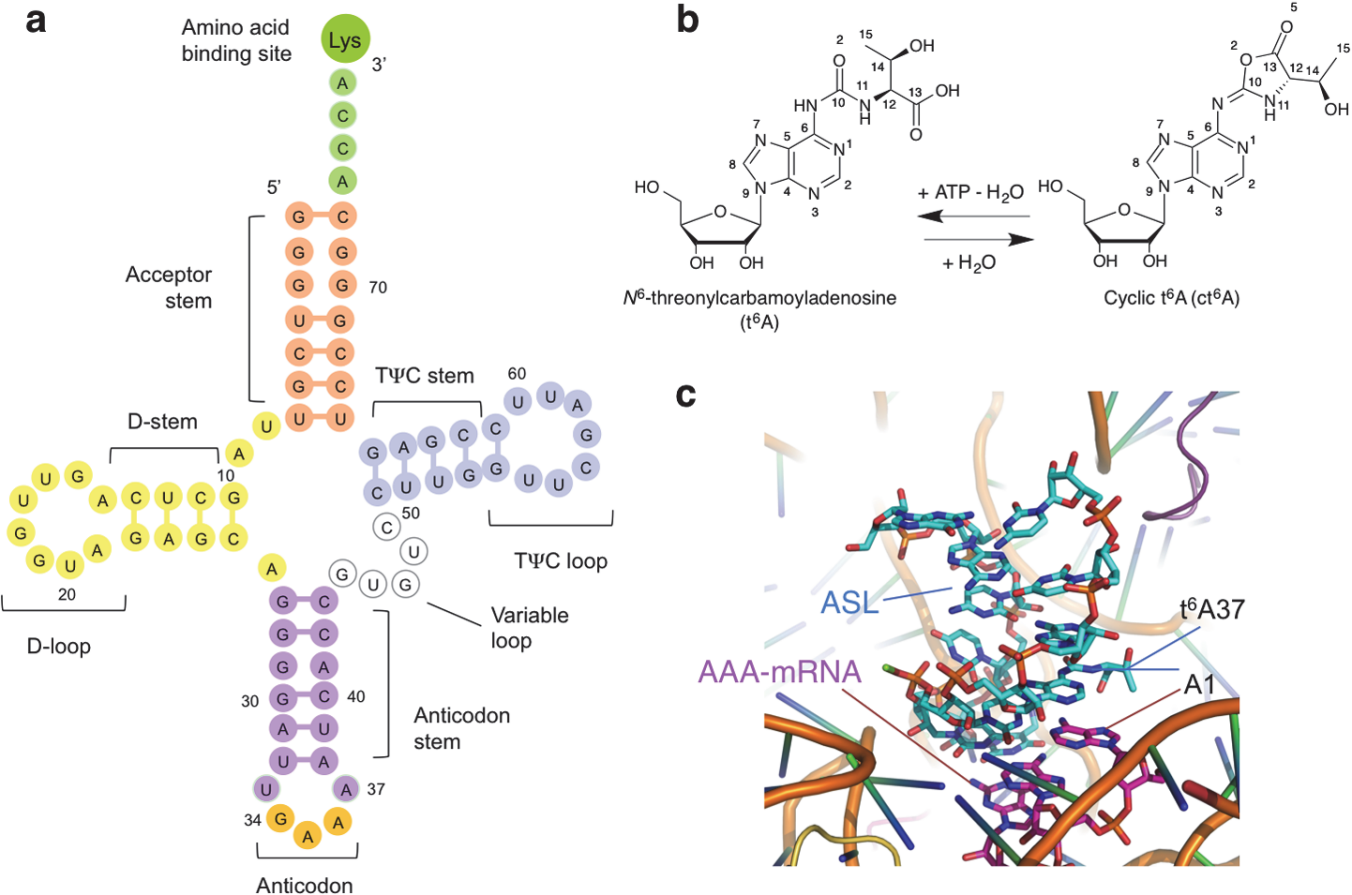
Cyclic N^6^-threonylcarbamoyladenosine (cyclic t^6^A, ct^6^A) hypermodification in tRNA. **a** Schematic cloverleaf representation of tRNA^Lys^UUU showing the location of the ct^6^A modification at A37. **b** TcdA catalyzes the ATP-dependent cyclization of t^6^A into ct^6^A in tRNAs with ANN anticodons in bacteria, yeasts, fungi and plants, which occurs mainly at the A37 position of the anticodon stem loop (ASL) of the tRNA molecule. (**c**) Recognition of the AAA codon on mRNA by ASL of tRNA^Lys^UUU involves interactions between t^6^A37 and A1 from the AAA codon (PDB 1XMO).

Transfer RNA (tRNA) molecules are targeted by more than 100 different enzymes that introduce a large number of diverse post-transcriptional modifications in tRNA nucleotides. This variety of modifications range from simple modifications at the base and/or at the 2′-hydroxyl of the ribose (including, among others, methylation, thiolation, deamination, and base isomerization) to more complex hypermodifications [1,2]. The functional roles of these modifications include the stabilization of the tertiary structure of tRNA, controling gene expression, and modulating the interactions between tRNA and protein factors from e.g., the translational machinery [3]. Accordingly, tRNA modifications are essential for proper and efficient protein translation in all domains of life. Modifications in the anticodon loop of tRNA are crucial for decoding. A well-known example concerns the wobble modifications that occur at the first codon-anticodon position (U34), which participate in the regulation of cognate and near-cognate tRNA [3,4]. Hypermodified purine bases are frequently found 3′ adjacent to the anticodon loop, at position 37 (A37) (Fig. 1A). Modifications at A37 have been associated with the stabilization of codon-anticodon interactions through base-stacking at the ribosomal decoding site.

*N* ^6^-threonylcarbamoyladenosine (t^6^A; Fig. 1B) is one of the 15 universally conserved essential modifications found in all three domains of life [2]. With the exception of bacterial initiator tRNA^fMet^, t^6^A occurs at position 37 (t^6^A37) of the anti-codon stem loop (ASL) of all tRNAs which recognize ANN anticodons [5,6]. Further modifications of t^6^A37 have been described; two tRNA species in *E*. *coli* have an *N* ^6^-methyl derivative of t^6^A (m^6^t^6^A) and a 2-methylthio derivative of t^6^A (ms^2^t^6^A) is found in mammalian tRNA^Lys^ [7]. The breadth of functions fulfilled by t^6^A37 in promoting the codon-anticodon interaction and accurate decoding by the ribosome spans aminoacylation of tRNAs [8], tRNA binding to the A-site codon [9,10], efficient translocation [11], reading frame maintenance, preventing errors in AUG start codon selection and read-through of stop codons [12]. Steric hindrance from the bulky structure of t^6^A impedes U33-A37 base pairing [13] so that the anticodon loop can assume its canonical U-turn structure [14]. The crystal structure of the codon-anticodon interaction at the A site of the 30S ribosomal subunit [15] has shown that the extended planar ring structure of t^6^A37 (formed by intramolecular hydrogen bonding of the threonine moiety with the adenine ring) allows t^6^A37 to stack with A38 in the anticodon loop and the first adenine base (A1) of the codon in the messenger RNA (mRNA) (Fig. 1C and S1 Fig.), thereby stabilizing the codon-anticodon interaction at the decoding site.

In bacteria, yeast, protists and plants the well-known t^6^A modification has been recently shown to be a chemical hydrolysis artifact generated during the handling and preparation of tRNA [16]. Extraction of tRNA under extremely mild conditions has led to the identification of ‘cyclic t^6^A’ (ct^6^A), a cyclized active ester with an oxazolone ring (Fig. 1B), as the bona fide hypermodification at position 37 of tRNA for ANN codons in those organisms [16]. The enzyme responsible for t^6^A cyclization in *E*. *coli* was identified by comparative genomic approaches and analysis of LC/MS data sets of genomic deletion strains (ribonucleotide analysis) [17] as CsdL, subsequently renamed to TcdA, for tRNA threonylcarbamoyladenosine dehydratase A [16]. TcdA is an ubiquitin-activating E1-like protein [18] with detectable homology to the adenylyltransferases MoeB and ThiF. Previous studies have established that TcdA interacts with CsdE, a SufE-like sulfur acceptor [19], which in turn interacts with the cysteine desulfurase CsdA [20]. Indeed, ct^6^A synthesis *in vivo* seems to depend on the presence of a functional CsdA-CsdE sulfur relay system since ct^6^A abundance is drastically reduced in tRNA pools extracted from Δ*csdE* (2%) or Δ*csdA* (12%) cells [16]. Accordingly, CsdA alone or in concert with CsdE has been shown to support the enzymatic modification of TcdA by sulfur incorporation [18]. The relation between TcdA and CsdA-CsdE remains enigmatic, however, because ct^6^A is a non-thiolated modification and the synthesis on a tRNA substrate of ct^6^A starting from t^6^A can be reconstituted *in vitro* with TcdA and ATP without CsdA or CsdE [16].

To start building a framework to investigate the molecular recognition processes underlying TcdA binding to ANN recognizing tRNAs and the structural and functional properties of TcdA responsible for the catalytic dehydration of t^6^A into ct^6^A, we set out to determine the structure of TcdA and its complex with tRNA^Lys^(UUU). Toward this end, we have obtained high-resolution crystal structures of TcdA in complex with AMP and ATP, which show that the E1-like enzyme fold of TcdA exhibits a unique architecture that requires K^+^ cations rather than Zn^2+^ for structural integrity (in contrast to most other E1-like proteins known) and a highly coordinated Na^+^ cation at the dimer interface. Structural characterization by solution small-angle X-ray scattering (SAXS) of the TcdA complex with tRNA^Lys^(UUU) reveals the position and relative orientation of the tRNA^Lys^(UUU) substrate bound to TcdA. Finally, we have also probed into the structural and functional connection of TcdA with the CsdA-CsdE sulfur relay system using nuclear magnetic resonance (NMR) methods since CsdA and CsdE are essential components for ct^6^A37 synthesis *in vivo* [16].

## Materials and Methods

### Recombinant TcdA production

*E*. *coli* TcdA (full length) was expressed in *E*. *coli* BL21 (DE3) cells harboring a C-terminal hexa-histidine tag. TcdA was purified using conventional nickel-chelating affinity chromatography (HisTrap, GE Healthcare) coupled to size-exclusion chromatography (Superdex 200 10/300GL, GE Healthcare) in 20 mM sodium/potassium phosphate, pH 7.4, 300 mM NaCl and 2 mM 2-mercaptoethanol.

### Preparation of tRNA

The gene for *E*. *coli* tRNA^Lys^(UUU) was cloned into the pET23a vector under the control of the strong T7 promoter, and expressed in *E*. *coli* BL21(DE3) cells grown for 3 h at 37°C after induction with 1 mM IPTG. The tRNA pool containing the over-expressed tRNA^Lys^(UUU) was extracted from the cells by resuspending the cell pellet (from 200 ml culture) using acid phenol (pH 4.3), precipitated with 100% ethanol, and was further purified by size exclusion chromatography over a Superdex 75 HR 16/60 column (GE Healthcare) in phosphate buffer saline (PBS), 0.1 mM EDTA, pH 7.4. All buffers used for extraction and purification of tRNA^Lys^(UUU) were twice autoclaved and the tRNA samples were kept at 4°C at all times to minimize the action of potential RNase contaminants. Agarose gel electrophoresis of the tRNA pool extracted from *E*. *coli* cells showed at least a 10-fold greater concentration upon induction when compared with non-induction controls, resulting in a tRNA pool highly enriched in recombinantly expressed tRNA^Lys^(UUU).

### Electrophoretic mobility shift assays with tRNA^Lys^(UUU)

Gel retardation assays of TcdA complexed with over-expressed tRNA^Lys^(UUU) or with commercial tRNA^Lys^(UUU) (Sigma-Aldrich R1753), were performed by electrophoresing pre-incubated complexes and appropriate controls on native 8% polyacrylamide-0.5× Tris-borate-EDTA (TBE) gels at constant current (12 mA) for 1 h at 4°C. Gel retardation assays with the two sources of tRNA^Lys^(UUU) gave identical results. Protein bands were stained with Coomassie Brilliant Blue and tRNA bands with Toluidine Blue.

### Analytical ultracentrifugation

Sedimentation velocity (SV) and sedimentation equilibrium (SE) analytical ultracentrifugation (AUC) experiments were conducted in a Beckman Coulter ProteomeLab XL-I analytical ultracentrifuge equipped with UV-Vis absorbance and Raleigh interference detection systems, using the 8-hole Beckman An-50Ti rotor at 20°C. TcdA, tRNA^Lys^(UUU) and TcdA-tRNA^Lys^(UUU) in 20 mM HEPES, 100 mM KCl and 50 mM NaCl, pH 7.4, were loaded (320 μl) into analytical ultracentrifugation cells. SV at 20 000 rpm was monitored by absorbance at 290 nm with scans made at 1 min intervals. Sedimentation coefficient distributions were calculated by least-squares boundary modeling of SV data using the continuous distribution c(*s*) Lamm equation model as implemented in SEDFIT 14.1 [21]. Experimental *s* values were corrected to standard conditions (water, 20°C, and infinite dilution) in SEDNTERP to derive the corresponding standard *s* values (*s*_20,w_). Short column (90 μl) SE experiments were carried out at speeds ranging from 8 000 to 12 000 rpm and monitored by absorbance at 280 nm, 290 nm and 295 nm. After the last equilibrium scans, a high-speed centrifugation run (48 000 rpm) was done to estimate the corresponding baseline offsets. Weight-average buoyant molecular weights of protein and nucleic acid species were determined by fitting a single species model to the experimental data using the HeteroAnalysis software [22,23] corrected for solvent composition and temperature with SEDNTERP [24].

### TcdA crystallization, structure determination and refinement

Crystals of TcdA (full length, as a C-terminal hexa-histidine fusion) in complex with ATP or as obtained directly from the cell (AMP) were grown at 5 mg/ml TcdA in presence of 0.05 M potassium phosphate and 10% PEG1000 at basic pH at 20°C. The construct of TcdA and the crystallization conditions used in this study are different to those used in a recent crystallization report [25]. Two X-ray diffraction data sets were collected from crystals of the ATP complex, one to a maximum resolution of 1.77 Å at a wavelength of 0.97948 Å, and a second data set to 2.35-Å resolution at a long wavelength (1.99976 Å) for sulfur single anomalous diffraction (SAD), at the BL13-XALOC beamline (ALBA, Barcelona, Spain). A data set for the AMP complex was collected to 1.89-Å resolution at the PROXIMA 2A beamline (Synchrotron SOLEIL, Paris, France). All the data sets were integrated with XDS [26] and scaled with Aimless [27] from the CCP4 suite of programs [28] (Table 1).

**Table 1:** Crystallographic data processing and refinement statistics.

	TcdA-ATP	TcdA-AMP
PDB code	4D79	4D7A
**Data collection**		
Wavelength (Å)	0.9795	0.9801
Resolution range (Å)	41.13–1.77 (1.83–1.77)	41.14–1.89 (1.96–1.89)
Space group	*P* 1 21 1	*P* 1 21 1
Unit cell dimensions		
*a*, *b*, *c* (Å)	65.3, 96.7, 82.8	65.7, 97.2, 83.2
β (°), α = γ = 90°	90, 111.2, 90	90, 111.6, 90
Total reflections	312,887 (11,351)	146,386 (14,469)
Unique reflections	89,883 (5907)	87219 (78261)
Multiplicity	3.5 (1.9)	1.9 (2.0)
Completeness (%)	95.96 (63.90)	97.14 (92.68)
Mean *I*/σ(*I*)	18.97 (3.75)	18.99 (3.13)
Wilson B-factor	23.95	24.01
R-merge	0.08388 (0.663)	02326 (0.6354)
R-meas^a^	0.09797	0.327
CC1/2^b^	0.995 (0.556)	0.878 (0.243)
CC*^c^	0.999 (0.846)	0.967 (0.625)
**Refinement**		
R-work	0.1416 (0.2994)	0.1396 (0.1839)
R-free	0.1833 (0.3236)	0.1768 (0.2350)
# non-H atoms	8167	8064
# Protein atoms	7484	7411
# Ligand atoms	172	46
# Water	511	607
Protein residues	996	974
RMS(bonds) (Å)	0.011	0.007
RMS(angles) (°)	1.430	1.01
Ramachandran analysis		
Favored/Allowed/Outlier (%)	98.0/2.0/0.0	98.0/2.0/0.0
Clashscore	1.88	1.93
Average *B*-factor (Å^2^)	34.40	32.10
Protein	33.60	31.50
Ligands	60.80	49.70
Solvent	38.00	37.20

^a^Rmeas = Σ_hkl_ (n/n-1)^1/2^ Σ_i_ |I_i_(hkl)-<I(hkl)>| / ΣΣ_i_ I_i_(hkl); where i is the ith measurement of reflection (hkl) and <I(hkl)> is the average over symmetry related observations of a unique reflection (hkl).

^b^CC1/2 is the Pearson correlation coefficient calculated between two random half data sets.

^c^CC* is the CC of the full data set against the true intensities, estimated from CC* = [2 CC1/2/(1+CC1/2)]^1/2^.

The structure of TcdA in complex with ATP was solved at 1.77-Å resolution using MR-SAD by first placing residues 21–199 of MoeB (PDB 1JWA) [29] with PHASER [30] using the high-resolution data set, and then using the partial model and the anomalous signal of sulfur, phosphorus (from ATP) and potassium measured on the long wavelength data set to phase the full length structure. The complete structure of TcdA could be built into the minimally biased electron density maps calculated from MR-SAD phases; the only exception is a solvent exposed loop spanning residues 217–236 above the ATP binding pocket. The structure of the AMP complex was solved by MR using the ATP complex as search model. Both structures were built, refined and validated with *Coot* [31], phenix.refine [32] and MolProbity [33].

### Accession codes

The atomic coordinates and structure factors for the determined crystal structures are deposited in the Protein Data Bank (PDB) under accession numbers 4D79 (TcdA-ATP) and 4D7A (TcdA-AMP).

### Fluorescence spectroscopy

The dissociation constant for the TcdA-tRNA^Lys^(UUU) binding interaction was obtained by following the quenching of the intrinsinc fluorescence of tryptophan (2) and phenylalanine (8) residues in TcdA upon tRNA^Lys^(UUU) addition. All fluorescence spectroscopy experiments (100 μl) were conducted in 20 mM sodium/potassium phosphate, pH 7.4, 300 mM NaCl, at 25°C using a Varioskan (Thermo Fisher) instrument, in black 96-well plates, setting the excitation and emission wavelengths to 280 nm and 340 nm, respectively. TcdA variants at 5 μM were challenged with increasing concentrations of tRNA (0.1–10 μM) in triplicate. Primary inner-filter effects due to tRNA absorption at the excitation wavelength were corrected for by standard methods (secondary inner filter effects for tRNA at 340 nm are negligible) and collisional induced quenching (as opposed to binding) was ruled out as the mechanism for the quenching of TcdA intrinsic fluoresence [34]. The reduction in fluorescence signal that accompanied tRNA addition was analyzed by non-linear regression methods in SigmaPlot v12, and the dissociation constant, *K*_D_, and the Hill coefficient were calculated according to the following equation:

$$\frac{F_{0}-F}{F-F_{c}}=\frac{L^{n}}{K_{D}+L^{n}}$$

where F_0_ is the intrinsic fluorescence of TcdA in the absence of ligand, F_c_ is the minimal residual fluorescence of the TcdA-tRNA^Lys^(UUU) complex and L is the tRNA^Lys^(UUU) concentration.

### NMR experiments

NMR experiments were acquired at 298 K on a Bruker Avance 600 MHz spectrometer equipped with a cryoprobe for 1D and 2D spectra, and on a Bruker Avance 800 MHz for the 3D. The spectra were processed with TopSpin 2.1 (Bruker), and 2D and 3D spectra were analyzed with the program CCPN Analysis [35]. 1D ^1^H-^13^C and ^1^H-^15^N HSQC spectra (5120 and 10240 scans respectively), 2D ^1^H-^15^N HSQC spectra (3072 data points in t2, 128 in t1, 96 scans), and 3D spectra (2048 data points in t3, 30 in t2 (^15^N), and 128 in t1 (^13^C), 32 scans) were acquired using standard Bruker pulse sequences. 1D heteronuclear spectra were processed with a line broadening of 10 Hz and 20 Hz (^1^H-^13^C and ^1^H-^15^N HSQC respectively). ^1^H-^15^N backbone resonance assignment for CsdE was carried out using HSQC, CBCANH [36] and CBCA(CO)NH [37] spectra and based on deposited data (BMRB code: 5630) [38].

Samples for the interaction between TcdA and CsdA were prepared as follows: a solution of 100 μM of ^13^C/^15^N-TcdA (in binding buffer, consisting of 20 mM sodium/potassium phosphate, pH 7.4, 300 mM NaCl, 2 mM dithiothreitol) was divided in two parts: 100 μl of one of them was diluted with 80 μl of binding buffer and 20 μl of D_2_O, resulting in a 50 μM solution of free TcdA. To the remaining 100 μl, 80 μl of a 1.3 mM solution of CsdA (unlabeled) and 20 μl of D_2_O were added, resulting in a 1:10 ratio solution TcdA:CsdA. 1D ^1^H-^13^C and ^1^H-^15^N HSQC spectra were acquired for both samples. 2D ^1^H-^15^N HSQC spectra were acquired for samples 0.09 mM of ^13^C/^15^N-CsdE containing 123 μl of binding buffer, and 0.09 mM ^13^C/^15^N-CsdE with 123 μl of 0.6 mM TcdA (ratio 1:4).

### Cross-linking experiments

The 7-atom, 8.0-Å short spacer arm cross-linking reagent bis(maleimido)ethane (BMOE) (Pierce 22322) generates non-cleavable cross-links between sulfhydryl groups that are in close proximity. BMOE was used to selectively trap transiently formed CsdE-TcdA complexes. CsdE and TcdA were mixed at a 1:1 molar ratio (80 μM) in reaction buffer (20 mM sodium/potassium phosphate, 450 mM NaCl, 0.5 mM EDTA, pH 7.4), treated with 1 mM TCEP for 30 min to break pre-existing unspecific disulfide bridges, and BMOE was then added to 0.2 mM final concentration and incubated for 1 h at 25°C. Control reactions containing either CsdE or TcdA were set up and treated identically. The extent and specificity of the BMOE cross-linking reaction was followed by SDS-PAGE. The cross-linking reaction could be scaled up to obtain milligram amounts of CsdE-TcdA complex for biochemical and structural analysis (Supplementary Information).

### SAXS measurements

SAXS experiments were performed at the BM29 BioSAXS beamline at the ESRF (Grenoble, France) [39]. SAXS data from purified TcdA were collected using a batch setup at three concentrations between 0.5–5 mg/ml, with ten successive time frames and 20 s exposures. For TcdA-tRNA^Lys^(UUU), SAXS data (1 s per frame) were collected from two identical experiments using an online size-exclusion chromatography (SEC) setup [40] after injecting 100 μl of 8-mg/ml complex on a Superdex 200 Increase column (GE Healthcare). Since the complex was prepared in presence of a molar excess of the tRNA^Lys^(UUU) component, this step allowed to obtain scattering data from both the protein-tRNA complex and the excess tRNA^Lys^(UUU) on a single experiment. All SAXS measurements were performed at 5°C in 20 mM sodium/potassium phosphate buffer, pH 7.4, 300 mM NaCl, 2 mM β-mercaptoethanol. Data were recorded using a 1 M PILATUS detector (DECTRIS) at a sample-to-detector distance of 2.7 m and a wavelength of 1.5 Å, covering the range of momentum transfer 0.020 < s < 0.5 Å^-1^. Data from the batch setup (TcdA) or from the two equivalent peaks from the replicated SEC-SAXS measurements [TcdA-tRNA^Lys^(UUU) and tRNA] were averaged, buffer subtracted and merged using the procedures outlined in Round et al. [40]. The radius of gyration (R_g_) was evaluated using the Guinier approximation [41] and also from the entire scattering curve using Porod’s law [42], and the pair-distance distribution function P(*r*) was calculated using GNOM [43]. Guinier plots of all SAXS data support monodispersity of the analyzed samples (S2 Fig.). The measured scattering curves were compared with the theoretical scattering curves of the macromolecular models using CRYSOL [44]. *Ab initio* shape restoration was performed using 10–20 independent runs of DAMMIF [45] followed by DAMAVER [46] to create the final *ab initio* shape. ScÅtter was used to calculate the molecular weights of the macromolecules and for the calculation of the fitting parameter *R*_SAS_, the small-angle scattering invariant V_C_ and the parameter Q_R_ [47]. The summary of SAXS statistics is given in S1 Table.

## Results and Discussion

### Crystal structure of TcdA

**Fig 2:**
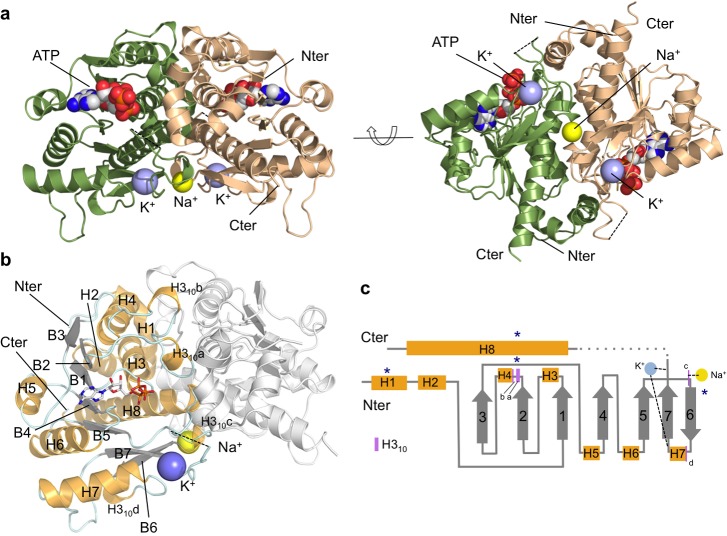
X-ray crystallographic structure of the binary complex TcdA at 1.77-Å resolution in complex with ATP. **a** Ribbon representation of the TcdA homodimer structure in two orientations related by a 90° rotation depicting each subunit in a different color (green or wheat). Two K^+^ and one Na^+^ cations are shown as violet and yellow spheres, respectively. The Na^+^ ion is located right at the homodimeric interface and its coordination sphere comprises amino acid residues from both monomers and water molecules. ATP is bound in a conserved surface pocket (shown as a space-filling model in CPK colors). **b** Description of secondary structural elements of TcdA monomer. Helices are shown in gold (α-helices are labeled H1-H8 and 3_10_ helices H3_10_a-d), strands in white (B1-B7), and loops in light cyan. **c** Annotated schematic representation of TcdA topology. Helices and strands are depicted as in **b**, except for 3_10_ helices which are indicated with purple lines and labeled a-d. K^+^ and Na^+^ ions are shown as blue and yellow circles. Dashed lines delimit regions that interact with K^+^, and structures involved in TcdA dimerization are marked with dark blue asterisks.

**Fig 3:**
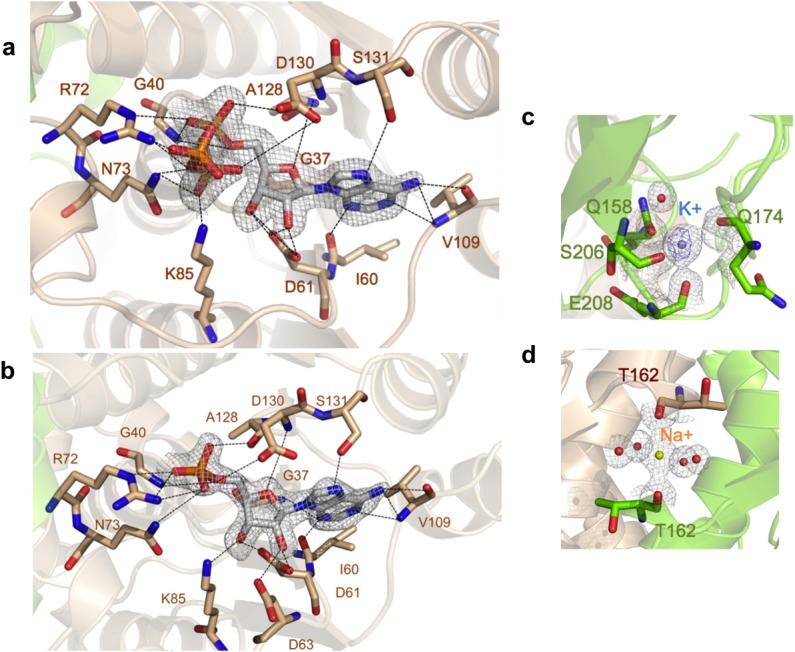
X-ray electron density maps of the ATP- and AMP-binding pocket and metal coordination spheres. **a** Detailed view of the ATP-binding site of TcdA. Residues that interact with ATP are labeled and shown as sticks and atom colors (C atoms are in subunit colors, as in Fig. 1). 2mF_o_-DF_c_ electron density map is depicted around the ATP substrate at 1.5 σ contour level. **b** Detailed view of AMP bound in the active site. Representation and electron density map as in (**a**). **c**, **d** Metal coordination spheres of K^+^ (**c**) and Na+ (**d**) cations. Interacting residues are labeled and shown as sticks in atom colors (C atoms are in subunit colors), and coordinating water molecules are shown as red spheres. Metals and their coordination spheres are shown in 2mF_o_-DF_c_ electron density (grey). The anomalous Fourier map calculated from a long wavelength (1.89 Å) dataset at 2.4-Å resolution is shown for K^+^ (**c**) in purple; in contrast, Na^+^ has no anomalous signal at that wavelength, enabling the accurate discrimination between K^+^ and Na^+^ cations in the structure.

The recent functional assignment of TcdA as the dehydratase in the synthesis of ct^6^A was not predictable from the primary structure, since it has detectable sequence homology only to the ubiquitin-activating E1-enzymes MoeB (14%) and ThiF (11%). These enzymes are unknown to bind tRNA or catalyze post-translation modifications in tRNA substrates. The similarity to MoeB/ThiF is restricted to the N-terminal domain of TcdA (residues 30–157), which is classified into the ThiF family (Pfam database PF00899; E-value 6.7 × 10^–41^) [48]. In contrast, the C-terminal end of TcdA lacks sufficient sequence similarity for functional prediction. To shed light onto the structural basis for the tRNA binding and ct^6^A synthetic properties of TcdA-ATP, we determined the crystal structure of *E*. *coli* TcdA (Table 1 and Fig. 2) loaded with ATP to 1.77 Å resolution (R/R_free_ values of 0.139/0.176) (Fig. 3A) and AMP to 1.89 Å resolution (R/R_free_ values of 0.141/0.183) (Fig. 3B). The asymmetric unit contained four TcdA chains arranged in two independent dimers, with a solvent content of 39%. Analysis of the macromolecular interactions and potential complexes in the crystalline state with PISA [49] indicates that the TcdA dimers are the most likely assembly in solution. The structure of TcdA in complex with ATP was solved by molecular replacement-single wavelength anomalous diffraction (MR-SAD) using as search model residues 21–199 of MoeB (PDB 1JWA) [29] and the anomalous signal coming from protein sulfur atoms, ATP phosphorus atoms and K^+^ cations as additional phasing information. The structure of TcdA-AMP was solved by MR using the fully refined model of the TcdA-ATP complex. Besides localized changes in the active site, the TcdA-ATP and TcdA-AMP structures are identical (RMSD 0.16 Å over 246 Cα atoms).

The tertiary structure of monomeric TcdA consists of seven β-strands in a continuous sheet surrounded by eight α-helices (Fig. 2). All β-strands are parallel except for the sixth β-strand (β6) at the end of the sheet (Fig. 2C). As in the MoeB/E1 superfamily, the N-terminal half of the sheet contains a variation of the Rossman fold whereby the βαβαβ-topology is interrupted between β2 and α4 by the insertion of two consecutive 3_10_ helices. The first 3_10_ helix (H3_10_a) contains three residues that are conserved in the MoeB/E1 superfamily, including a strictly conserved Asn69 that interacts with bound ATP/AMP via water-mediated hydrogen bonds. In agreement with its ATP hydrolytic activity, the loop between β1 and α3 contains the highly conserved Gly-X-Gly-Ala/Gly-Leu/Ile-Gly motif (where X denotes any residue, typically Val, Leu and Ile), akin to the P loop [50]. The C-terminal half of TcdA comprises three parallel β-strands (β4, β5 and β7) hydrogen bonded to the antiparallel β6, two α-helices connecting consecutive β-strands (α6 and α7), and a long α-helix (α8) that runs perpendicular to the TcdA dimer symmetry axis and sticks out on either side (Fig. 2). Several of the C-terminal motifs including β6, α7 and β7 and the loops connecting them generate two unique metal binding sites, a K^+^ binding site per TcdA monomer and the interfacial Na^+^ binding site (Fig. 2).

In the TcdA-ATP complex (Table 1 and Fig. 3A), ATP is bound in a cleft over the C-terminal end of the central sheet. The residues in the P loop form the floor of the nucleotide triphosphate moiety, and the adenine ring is inserted into a hydrophobic cavity. Specific interactions anchor ATP at the active site through its triphosphate moeity and the ribose hydroxyl groups. A short hydrogen bond between the main-chain carbonyl oxygen from the P-loop Gly40 maintains the α-phosphate group firmly bound insite the binding pocket in a kinked conformation. The conserved Arg72 and Lys85 contact oxygens from the α- and β-phosphates, while Thr68 and the strictly conserved Asn69 in the H3_10_a helix anchor the γ-phosphate through a water-mediated hydrogen bond. In the TcdA-AMP complex (Table 1 and Fig. 3B), the nucleotide-binding residues stabilize AMP in a manner analogous to ATP, with no conformational changes in the TcdA dimer. In the TcdA-ATP and TcdA-AMP complexes, the region between β7 and the C-terminal α8 spanning residues 214–236 is fully disordered in electron density maps. This surface disordered loop, in close proximity to the ATP binding site, corresponds to the disordered loop regions of MoeB [29] that are thought to play catalytic roles or engage in protein-protein interactions.

One K^+^ binding pocket is found deeply buried in each TcdA monomer, as supported by anomalous Fourier maps of the K^+^ ions contoured at 7 σ (Fig. 3C). The geometry of the coordination sphere of K^+^ is distorted tetrahedral, and the ligand residues include main-chain carbonyl oxygens (Gln174, Ser206 and Glu208) and the side chain of Gln158, with an average metal-oxygen distance of 2.7 Å, and a water molecule at 2.6–3.1 Å. The K^+^ binding site is created by structural elements in the C-terminal half of TcdA comprising the loop β6-α7, β7 and loop-β7. This unique surface pocket is absent in other members of the MoeB/E1 superfamily and could have functional roles specific to tRNA binding and/or modification.

A second metal binding site specific for Na^+^ is found at the dimer interface in an octahedral coordination (Fig. 3D). The Na^+^ cation is axially liganded by the carbonyl oxygens of two symmetric Thr162 residues that lie in a short 3_10_ helix segment (H3_10_c) from opposite monomers, with four water molecules occupying the equatorial positions. The identity of this sodium ion was unambiguous owing to the conspicuous coordination sphere of sodium, with average coordination distances of 2.35 Å between the metal and the carbonyl oxygens, and 2.50 Å with the water oxygens, and the total absence of anomalous signal at 1.999 Å, which ruled out a K^+^.

### Dimeric structure of TcdA

**Fig 4:**
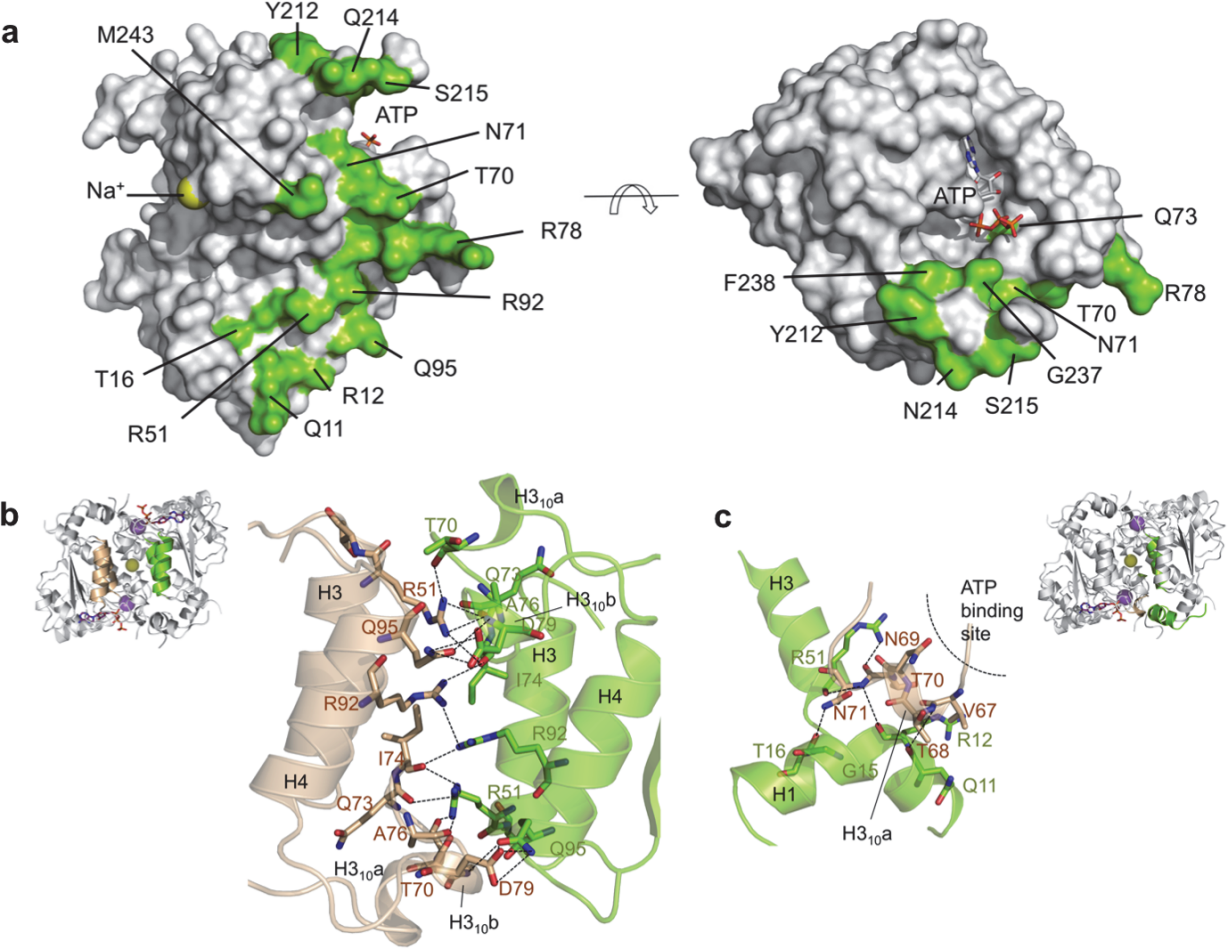
TcdA dimerization interface. Molecular surface representation of a TcdA dimer in two orientations (**a**) related by a 90° rotation around a horizontal axis. The location of the ATP binding pocket and the interfacial Na^+^ ion are labeled. Amino acid residues that contribute to the extended, flat dimer interface are mapped onto the molecular surface in green and labeled. **b** Close-up of the symmetric helical bundle (H3-H4). Key hydrogen bonding and charge interactions are shown as dashed lines. **c** Helix H1 participates in the dimer interface through hydrogen bonding and van der Waals interactions with H3 from the same chain and H3_10_a from the opposite monomer.

The overall architecture of TcdA consists in a symmetric dimer, consistent with the observed elution volume of TcdA in analytical gel filtration (not shown) and solution SAXS scattering data (S1 and S2 Tables). The TcdA dimer is maintained by Na^+^, which mediates monomer-monomer contacts through the dimer interface, and by K^+^ ions, which play a structural role in shaping the monomer fold. The dimer interface buries approximately 2250 Å^2^ solvent accessible surface area (ASA), or 20% of the total ASA per monomer, engaging >25% of TcdA residues along an elongated surface patch (Fig. 4A). According to PISA [49], the TcdA dimer is chemically stable as its free Gibbs energy of dissociation is 21.5 kcal/mol. This surface patch comprises four helices (α1, α3, α4 and the long C-terminal helix α8) from each monomer, the 3_10_ helical regions H3_10_a and H3_10_c that shape the nucleotide-binding site and the Na^+^ binding site, respectively, and the loops β5-β6 and β7-α8. In addition to many hydrophobic interactions, several strong hydrogen bonds stabilize the dimer structure, engaging interfacial arginine residues from helices α1 (Arg12), α3 (Arg51), H_10_b (Arg78) and α4 (Arg92) (Fig. 4B,C). Two symmetric interactions further contribute to the dimer stability, the antiparallel interaction between helices α8 and the Na^+^-bridged interaction held by Thr162 main-chain oxygens (Fig. 3D).

### Comparison with MoeB/E1 superfamily

**Fig 5:**
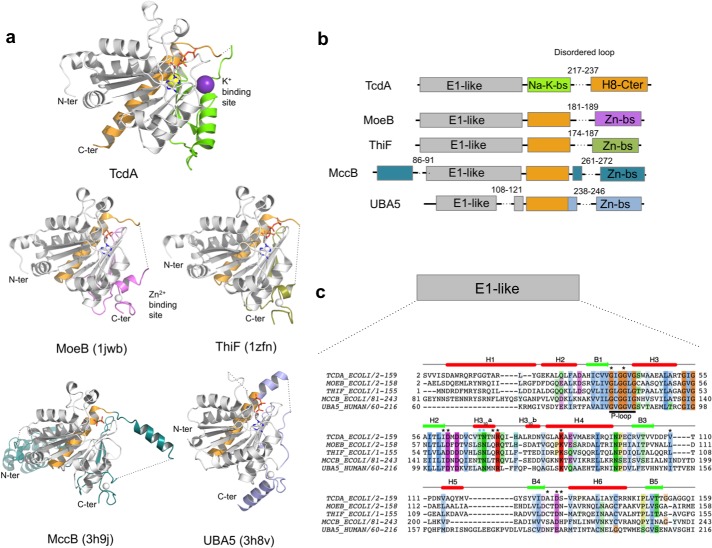
Structural comparison of TcdA with the homologous E1-like activating enzymes. **a** Ribbon representation of TcdA structural homologs found with PDBeFold [51] with structural similarity Q-scores higher than 0.28 (the highest Q-scores are 0.49 with MoeB and 0.46 with ThiF). All structures were superimposed onto TcdA and are shown in similar orientations for comparison: MoeB (PDB 1JWB), ThiF (PDB 1ZFN), MccB (PDB 3H9J) and UBA5 (PDB 3H8V). E1-like core domain is shown in grey; the long α-helix that is structurally equivalent to TcdA α8 is in orange; metal binding sites are in protein-specific colors; K^+^ ions are in purple, Na^+^ ions in yellow, and Zn^2+^ in grey. **b** Schematic representation of the domain architecture of the E1-like enzymes superimposed in (**a**), with equivalent color coding; bs, binding site; the sequence position corresponding with disordered loops is indicated by dashed lines and marked with start and end residues. The C-terminal α8 helix in TcdA is structurally equivalent to the α-helix immediately following the E1-like domain in the other enzymes. **c** Structure-guided multiple sequence alignment of the E1-like domain with overlaid secondary structure from TcdA. Functionally important residues and motifs are annotated as follows: bold underline, P-loop residues; black asterisks, residues in direct contact with ATP; and blue asterisks, residues from helix H3_10_a that make water-mediated contacts with the nucleotide. Conserved residues are shown in shaded colors.

There is significant structural similarity between the 180-amino-acid N-terminal half of TcdA and the catalytic domain of MoeB/ThiF, which can be superimposed with RMSD of 1.57 Å (181 Cα atoms) and 1.42 Å (177 Cα atoms), respectively (Fig. 5). The MoeB/E1 superfamily enzymes *E*. *coli* MccB and human UBA5, which share the same fold structure than MoeB/ThiF, also display a considerable degree of structural similarity to TcdA, with RMSD of 1.74 Å (173 Cα atoms) and 1.85 Å (146 Cα atoms), respectively (Fig. 5). The nucleotide-binding pocket and a significant part of the helices that in TcdA participate in the dimer interface are among the most conserved structures between TcdA and MoeB/ThiF. In particular, the P loop and the residues that contact ATP/AMP are nearly strictly conserved across all structural homologs.

In contrast to the conservation displayed by the nucleotide-binding site and adjacent regions, the C-terminal half of TcdA adopts a unique structure that is distinct from those present in MoeB/ThiF. Firstly, the C-terminal extension of TcdA is stabilized by a structural K^+^ cation and not by Zn^2+^, which is consistent with the absence in TcdA of the Cys4 motif responsible for Zn^2+^ binding in MoeB/ThiF. Of the four cysteine residues used by MoeB/ThiF to chelate Zn^2+^, TcdA has retained only the last cysteine (Cys220), while the first two cysteine residues fall in a sequence stretch absent in TcdA and the third cysteine is a threonine in TcdA (Thr218). Cys220 is situated in the beginning of the disordered loop and therefore could have a functional role during catalysis. Cys234 in TcdA is also conserved in MoeB/ThiF, and it is found in the disordered loop. In contrast, an additional Cys66 without an equivalent residue in MoeB/ThiF is located near the active site (S3 Fig.). Since functional thiol transfer from CsdA-CsdE appears to be essential for TcdA function *in vivo* [16], and thiolation of TcdA has been shown to occur *in vitro* [18], it seems likely that Cys66, Cys220 or Cys234 may be susceptible to be the recipient of the activated sulfur atom.

As a result of the structural differences in the C terminus of TcdA and MoeB/ThiF, the solvent-exposed surface motifs that allow the MoeB/MoeD and ThiF/ThiS complexes to form are blocked in TcdA by an extended segment containing residues 170–189, which is part of the K^+^ binding site. This motif is the only structural element that has no counterpart in MoeB and ThiF, raising the possibility that it is involved in TcdA specific functional roles absent in other E1-like enzymes. In addition, structure superposition of TcdA with MoeB, ThiF, MccB and UBA5 (Fig. 5 and S4 Fig.) shows a difference in domain organization consisting in a swap between the long helix (orange) and the metal binding sites. In TcdA, in contrast to all other structural homologs, the metal binding site occupies a central position between the E1-like domain and the C-terminal α8.

### TcdA can establish transient interactions with CsdE and CsdA

Previous studies have established that TcdA can be persulfurated by the cysteine desulfurase CsdA, either directly or indirectly through the SufE-like sulfur acceptor CsdE [18], and that the *csdA* and *csdE* genes are essential to support the function of TcdA in ct^6^A biosynthesis *in vivo* but not in an *in vitro* reconstituted system [16]. To shed light into the proposed interaction between TcdA and CsdA-CsdE we set out to characterize their complex. We were not able to identify a stable CsdA-TcdA or CsdE-TcdA complex by gel filtration or pull-down experiments (data not shown) even though a CsdE-TcdA complex had been previously isolated using overexpressed TAP-tagged CsdE [18]. To investigate whether a short-lived complex of TcdA with either CsdE or CsdA could form *in vitro*, we resorted to 1D and 2D HSQC NMR techniques that can detect fast exchanging transient protein interactions with high sensitivity.

To probe the hypothetical interaction between CsdA and TcdA, which would result in a 148-kDa heterotetramer (assuming one TcdA monomer bound per subunit of CsdA homodimer), we acquired 1D ^1^H-^13^C and ^1^H-^15^N HSQC spectra of 50 μM ^13^C,^15^N-TcdA (20% D_2_O) upon addition of unlabeled CsdA at a 10-fold excess molar ratio. Comparison of the resultant spectrum with a blank experiment recorded for only free isotope-labeled TcdA permitted to detect a decrease in the signal intensity of the TcdA resonances in the presence of ten-fold CsdA (data not shown). This decrease in intensity suggests the existence of a very weak interaction between CsdA and TcdA, consistent with published data reporting a catalytic interaction for sulfur transfer between TcdA and CsdA and the absence of a stable CsdA-TcdA complex by a yeast two-hybrid analysis [18].

**Fig 6:**
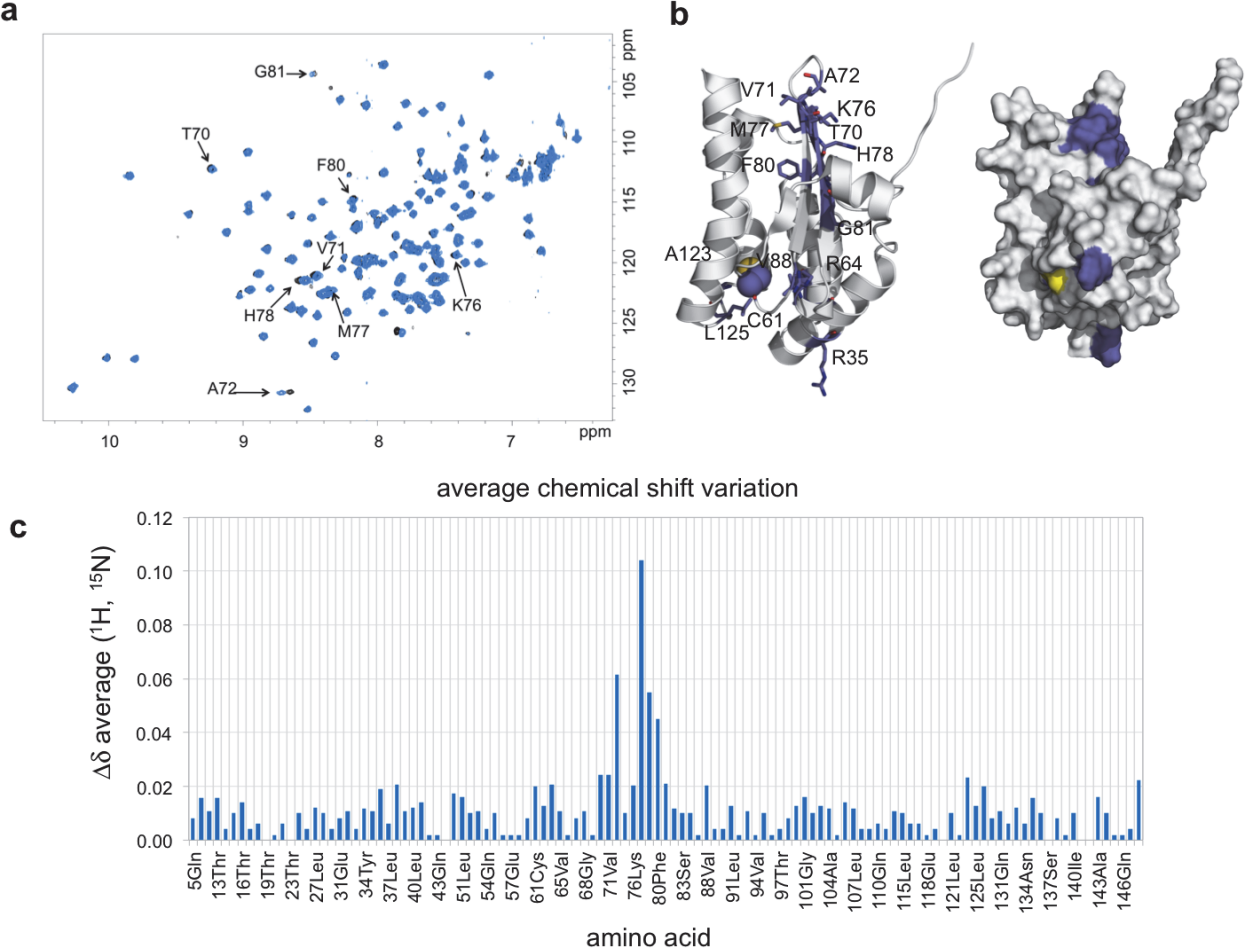
TcdA interacts transiently with the sulfur-acceptor SufE-like protein CsdE. **a** NMR ^1^H-^15^N NOESY spectrum of doubly labeled ^13^C,^15^N-CsdE in presence (blue) or absence (black) of unlabeled TcdA. Significant shifts in the position of specific NOESY resonance peaks for CsdE residues are labeled. **b** The position of the affected amino acid residues is mapped onto the NMR structure of CsdE (PDB 1NI7) in ribbon (left) and surface (right) representations. **c** Average chemical shift (Δδ) of ^13^C,^15^N-CsdE upon binding to TcdA as measured from the NOESY spectrum in **a** plotted against the CsdE amino acid sequence.

In contrast to the CsdA-TcdA complex, the smaller size of CsdE and the availability of its solution structure by NMR (bmr5630, PDB code 1NI7) [38] afforded the opportunity to further characterize the region of its putative interaction with TcdA. In this case, regular ^1^H-^15^N 2D-HSQC experiments (Fig. 6A) using ^13^C,^15^N-doubly labeled CsdE and native TcdA were performed. In these experiments, the ^1^H-^15^N HSQC spectra of free ^13^C,^15^N-CsdE (90 μM) and of a similar sample with 4-fold molar excess of unlabeled TcdA were acquired. The comparison of these spectra showed that several CsdE crosspeaks suffered chemical shift perturbations in the presence of TcdA, while most of them remained unperturbed. These evidences demonstrate the existence of a transient physical interaction between CsdE and TcdA. Given the observed chemical shift perturbations, the molecular recognition process is in fast exchange in the NMR chemical shift time scale, with an estimated *K*_D_ in the mM to high μM range. Under the low excess ratio conditions (1:4) between the components, only a small fraction of CsdE is probably bound in the CsdE-TcdA complex. Thus, the existence of a fast exchange process permits explaining the absence of complete signal decay. The assignment of the crosspeaks that were perturbed by TcdA allowed mapping the CsdE surface in contact with TcdA (Fig. 6B,C). In fact, the most perturbed region was located at residues 70–81, corresponding to the antiparallel hairpin that follows Cys61, the persulfurated Cys in CsdE, allowing its identification as the most crucial for TcdA interaction (Fig. 6C). This region has been characterized as being rather dynamic, as shown by both the CsdE and CsdA-CsdE crystal structures [38,52]. Very probably, the elongated structure of CsdE can facilitate further intermolecular interactions with the surface-exposed cysteine residues in TcdA.

To seek further support for a transient CsdE-TcdA complex, we performed cross-linking experiments with bis(maleimido)ethane (BMOE), a compound that cross-links sulfhydryls group that are in close proximity. The short 7-atom spacer arm of BMOE spans an 8.0-Å distance between the two cross-linked thiol groups, therefore enabling the capture of specific protein complexes. Our cross-linking experiments demonstrated the selective cross-linking of CsdE and TcdA in SDS-PAGE, with no cross-linked species being detected for CsdE or TcdA alone. The cross-linked CsdE-TcdA complex was stable against incubation with DTT and could be separated from excess components by gel filtration. The analysis of the cross-linked complex by SAXS revealed conformational flexibility that could be interpreted in terms of two independent CsdE molecules covalently attached to TcdA in pseudo-symmetrical positions (S1 Text and S5 Fig.).

Highly specific cross-linking of CsdE and TcdA with BMOE lends further support to the notion that CsdE and TcdA might engage in a complex. These weak, fast exchanging interactions may be functionally important for TcdA function provided that TcdA post-translational modification by persulfuration are required for proper function in ct^6^A biosynthesis.

### TcdA forms a complex with tRNA^Lys^

**Fig 7:**
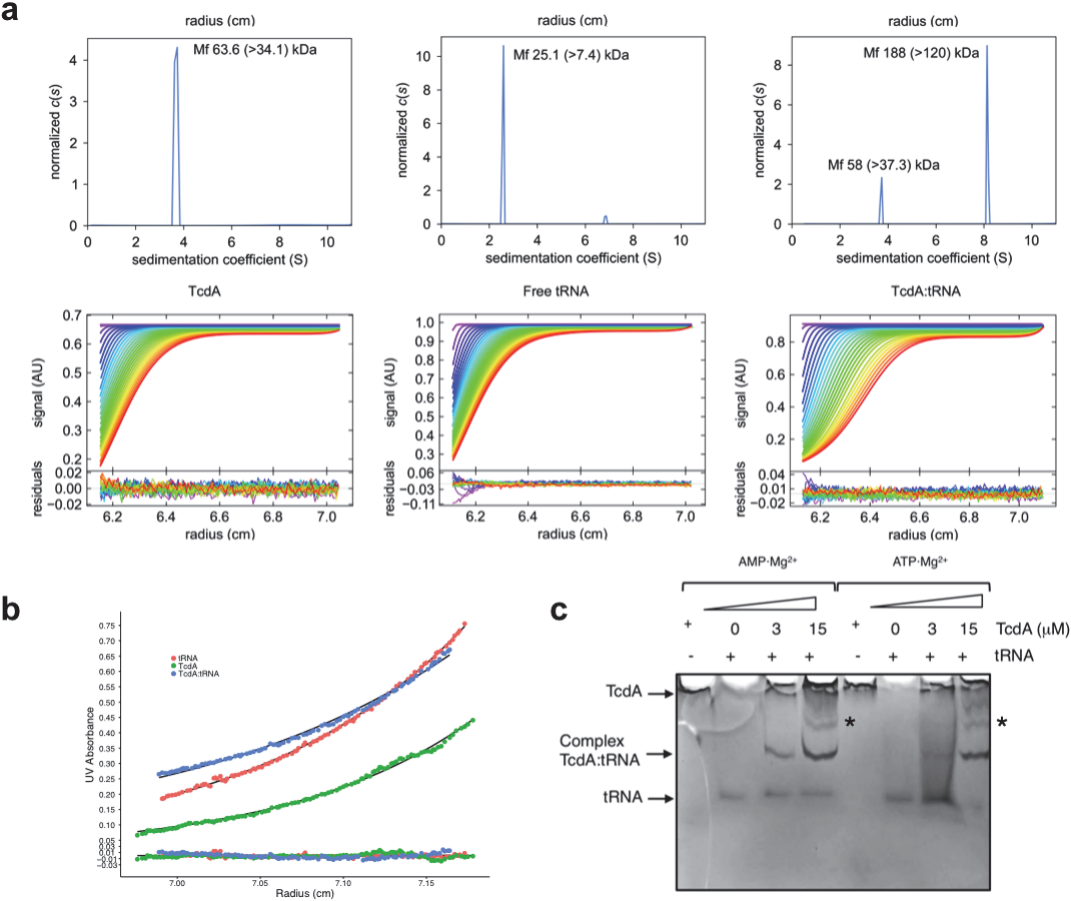
TcdA binding to tRNA. **a** SV-AUC experiments demonstrate that TcdA contains a tRNA^Lys^ binding domain. Sedimentation coefficient (S) continuous distributions, c(S), plotted for TcdA, tRNA^Lys^, and the TcdA-tRNA^Lys^ complex. Representative sedimentation scans and residuals after fitting are shown. For each c(S) distribution, the estimated molecular mass and the smallest mass compatible with the experimental results are indicated. **b** Absorbance at 280 nm plotted against the AUC column length for SE-AUC experiments for TcdA, tRNA^Lys^, and TcdA-tRNA^Lys^. Residuals after fitting are also shown. **c** Electrophoretic mobility shift assay (EMSA) provides evidence for the physical binding of TcdA to tRNA^Lys^. Samples were run over a 8% TBE-PAGE gel at 4°C for 1 h to fully resolve tRNA^Lys^ from TcdA-tRNA^Lys^ bands. TcdA does not enter the gel when tRNA^Lys^ is not added, most likely due to its intrinsically basic isoelectric point, pI (theoretical pI = 8.8). tRNA^Lys^ alone migrates as the fastest migrating band. When 5 M tRNA^Lys^ is added to 3 μM or 15 μM TcdA, protein-tRNA^Lys^ complexes develop that migrate behind free tRNA^Lys^. An arrow indicates the position of the retardation band containing the most abundant TcdA-tRNA^Lys^ complex (2:2 stoichiometry), and an asterisk marks the position of a slower migrating TcdA-tRNA^Lys^ complex (most likely, 2:1 stoichiometry).

The proposed role for TcdA in the transformation of t^6^A37 into its cyclic analogue, ct^6^A37, at position 37 of tRNA^ANN^, requires that TcdA engage in a productive binding interaction with its cognate tRNAs. Since no previous evidence for such a complex was available, we conducted electrophoretic mobility shift assays (EMSA) with purified TcdA and tRNA^Lys^(UUU) in the presence of the co-substrate Mg^2+^·ATP (Fig. 7C). These assays revealed the formation of a TcdA-tRNA^Lys^(UUU) complex that migrated as a distinct band when compared with TcdA alone (which did not enter the gel owing to its basic pI of 8.8) or isolated tRNA^Lys^(UUU), which migrated at a lower apparent molecular size. At the highest concentration of TcdA (15 μM), additional higher molecular weight bands become discernable, which may represent higher-order complexes. Although the physiologic quaternary structure of the TcdA-tRNA^Lys^(UUU) complex remains unknown, the dimeric structure of TcdA suggests that the fastest migrating complex retardation band may contain two tRNA^Lys^(UUU) molecules in a 2:2 stoichiometric complex (Fig. 7C, arrow) while the next slower migrating band might contain one tRNA^Lys^ molecule in a 2:1 substoichiometric complex with TcdA (Fig. 7C, *). The *K*_D_ for the TcdA-tRNA^Lys^(UUU) complex was determined as 3 μM by using the fact that the tryptophan fluorescence of TcdA is quenched by tRNA.

We further characterized the size and stability of the TcdA-tRNA^Lys^(UUU) complex by performing sedimentation velocity (SV) and sedimentation equilibrium (SE) analytical ultracentrifugation (AUC) experiments using complexes assembled in presence of a molar excess of the tRNA^Lys^(UUU) component. SV-AUC profiles for the complex was consistent with the formation of a high-molecular weight protein-tRNA complex that sedimented with a sedimentation coefficient of 8.2 S, compared with the isolated components (3.8 S for TcdA and 2.4 S for the tRNA) and with remaining free TcdA in the complex experiment (3.8 S) (Fig. 7A). Using the same experimental conditions for the previous experiments, we then ran SE-AUC for the TcdA-tRNA^Lys^(UUU) complex to measure the buoyant mass of the complex and assess the coexistence of excess individual components (Fig. 7B). The calculated molecular masses for the sedimenting species are within experimental error to the theoretical masses for all components: 63,531 ± 840 Da (TcdA dimer, 57.9 kDa), 26,066 ± 156 Da (tRNA, 23.4 kDa), and 114,297 ± 824 Da [TcdA-tRNA^Lys^(UUU), 104.8 kDa] (S2 Table).

### SAXS reconstruction of TcdA-tRNA^Lys^ complex

**Fig 8:**
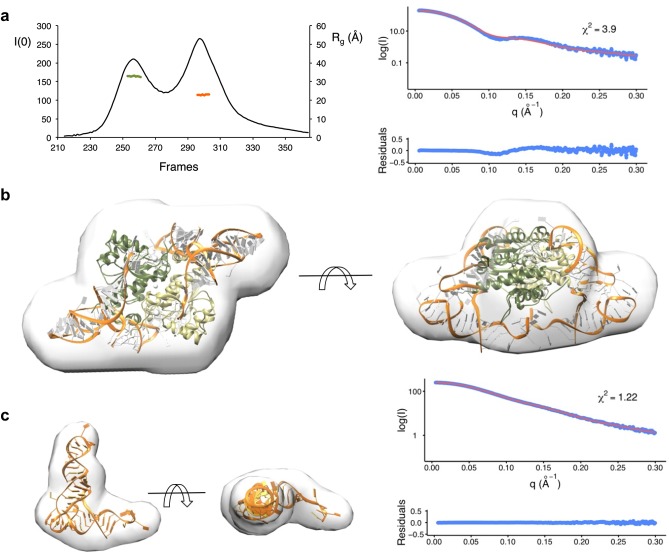
Solution structure of TcdA-tRNA^Lys^(UUU). **a** SAXS data for TcdA-tRNA^Lys^ was measured using an online HPLC setup to separate complex from excess free tRNA. The graph shows a plot of the SAXS intensity at zero angle, I(0) (left axis, curve represented as a solid black line), and of the radius of gyration, R_g_ (on the right axis), versus data-collection frames. Frames 253–263 (green line) were merged and used for shape restoration of the TcdA-tRNA^Lys^(UUU), and frames 304–310 were used for the control reconstruction of the tRNA^Lys^(UUU) shape. **b** Best model calculated for the TcdA-tRNA^Lys^(UUU) complex overlayed by the ab initio SAXS shape calculated with DAMMIF. The crystal structure of TcdA-ATP is represented in green cartoon and the tRNA is depicted with its main chain as a gold ribbon and the bases as ladders. The fit (red line) to the experimental SAXS data (blue points), calculated with CRYSOL, χ^2^ and residuals are shown. **c** Like in (**b**), for free tRNA^Lys^(UUU). In this case, the model is a rigid-body fit of the tRNA structure into the ab initio SAXS envelope.

In the absence of a crystallographic structure for the TcdA-tRNA^Lys^(UUU) complex, and in view of the dynamic nature of the complex as inferred from gel filtration and AUC experiments, we resorted to shape restoration by SAXS to elucidate the three-dimensional structure of the TcdA-tRNA^Lys^(UUU) complex. To collect X-ray scattering data from a monodisperse, homogenous solution of the complex, and circumvent the dynamic dissociation of the complex into its constituents, we subjected pre-incubated complex formed from purified components to on-line HPLC coupled with SAXS. The setup allowed us to collect useful solution scattering data from both the complex and from the excess tRNA^Lys^(UUU) (Fig. 8A).

Seven to fifteen successive frames across the non-overlapping peaks from a duplicated experiment containing TcdA-tRNA^Lys^(UUU) or free tRNA^Lys^(UUU) were processed and merged together on the basis of a coherent experimental R_g_ (R_g_ complex: 33.4 Å [Guinier points 24–53; sR_g_ 0.471–0.930; Fidelity 0.706]; R_g_ free tRNA: 23.0 Å [Guinier points 5–72; sR_g_ 0.139–0.892; Fidelity 0.979]). SAXS invariants were calculated for these species (reported in S1 Table). Furthermore, the estimation of the molecular weight using either the SAXS data (MW_SAXS_) or the AUC (MW_AUC_) data supports that the TcdA-tRNA^Lys^(UUU) complex contains a TcdA dimer and two tRNA molecules in solution (MW_SAXS_: 90.0 kDa; MW_AUC_: 114 kDa; compare with the theoretical MW for a 2:2 complex of 104 kDa) (S2 Table). In addition, SAXS data were collected also for TcdA, validating the crystallographic structure and permitting a more direct comparison of the complex to its components (S1 and S2 Tables).

*Ab initio* shape restoration from the SAXS scattering data was then used to generate the molecular envelope for the complex (Fig. 8B) and the free tRNA (Fig. 8C) with DAMMIF [45] and MONSA [53,54]. MONSA performs multiphase bead modeling for complexes with different contrasts but requires that the component structures do not undergo conformational changes in the complex. Preliminary analysis of the restored shapes suggested that conformational changes in TcdA were likely to be necessary for productive binding, therefore we used DAMMIF for all subsequent analysis. Since the TcdA-tRNA^Lys^(UUU) is two-fold symmetric, we generated bead models imposing either *P*1 or *P*2 symmetry. In both cases the resultant models were similar, but the shapes obtained with *P*2 symmetry reproduced better the symmetry known to be present in TcdA homodimer and were kept for further analysis. The overall shape of the TcdA-tRNA^Lys^(UUU) retrieved by SAXS contains a central body accounting for about 75% of the volume and two symmetric protrusions that extend to either side normal to the symmetry axis that we identified as tRNA^Lys^(UUU) (Fig. 8B).

**Fig 9:**
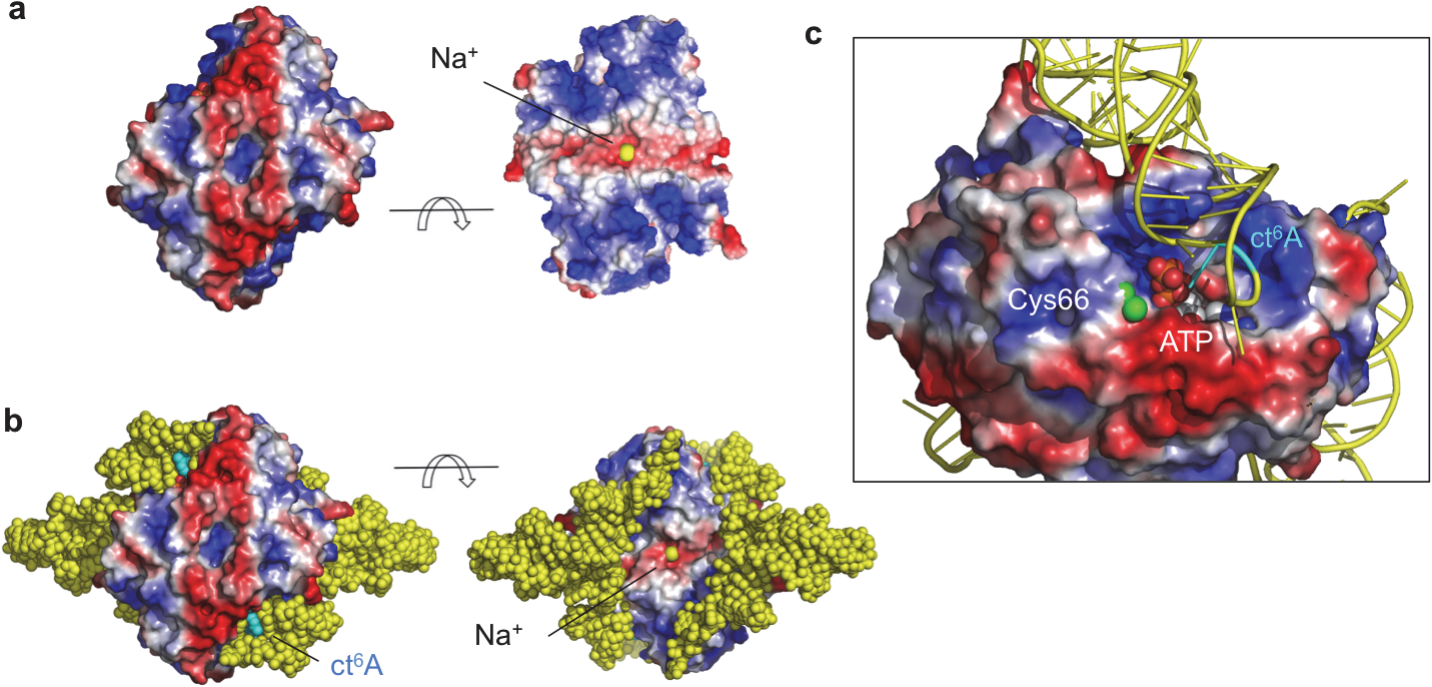
TcdA-tRNA interface. **a** Electrostatic potential molecular surface calculated with APBS (Adaptive Poisson-Boltzmann Solver) [55] and rendered with PyMOL (*www.pymol.org*) [56]. Two views are shown that are related by a 90° rotation around a horizontal axis. The interfacial Na^+^ cation is depicted as a yellow sphere. **b** tRNA is modeled on the basis of the SAXS data for the TcdA-tRNA^Lys^(UUU) complex bound to the two outer rims of TcdA (represented as in **a**), where most of the positively charged surface is found. The two tRNA molecules bind to spatially separated and independent surface patches in a symmetric arrangement. The modified ct^6^A37 nucleotide is shown in cyan. **c** Detailed view of the TcdA-tRNA binding mode. TcdA is represented as in (**a**), with Cys66 sulfur atom shown as a green sphere. The tRNA molecule on the front inserts its anticodon-stem loop into the ATP-binding pocket (in spheres and CPK colors), with ct^6^A37 (cyan) facing the catalytic site.

To gain insight into the structure of TcdA-tRNA^Lys^(UUU) we attempted to model the complex into the SAXS envelope, using the structure of TcdA-ATP and a representative structure of tRNA (PDB 4JXX). The shape and volume of the SAXS envelope indicates that TcdA could occupy a central location in the complex with two tRNA molecules symmetrically bound on either side of the complex (Fig. 8B), involving extensive contacts with the positively charged surface of TcdA (Fig. 9). Sequential or simultaneous rigid body fitting of TcdA-ATP and tRNA was possible with minimal intermolecular clashes, achieving a good description of the data (χ^2^ 2.3; *R*_SAS_ 0.001) (Fig. 8B). The SAXS-based model of TcdA-tRNA complex that emerges (Fig. 8 and 9) reveals how the flat surface beneath the dimer interface and the surface motifs involved in organizing the K^+^ binding site, which have been extensively modified from the E1-like enzyme primitive fold, play crucial roles in binding tRNA. This flattened interface provides an ideal docking platform for the flat side of the tRNA molecules while guiding the stem loop to position A37 in close proximity to the TcdA active site, and its electrostatic properties, complementary to those of tRNA, provide substantial binding stabilization. The large and symmetric electropositive surface patches on the TcdA structure located at the outer rims of the complex, which extend up and including the ATP binding site, provide an extensive positively charge surface for tRNA binding. Projecting the electrostatic potential surface derived from the crystallographic structures onto the shapes restored by SAXS further confirms that the TcdA surfaces involve in tRNA binding are also the most electropositive surface patches in the complex. It is then conceivable that a ternary TcdA-tRNA^ANN^ complex may form as an intermediate toward ct^6^A37 modification from which ATP hydrolysis and t^6^A37 cyclization steps may take place subsequently.

Although accurate modeling of the tRNA modification reaction within the TcdA-tRNA complex is impeded by the lack of high-resolution structures for the complex, the SAXS-based model of the complex is compatible with the known biological function of TcdA, whereby the modified t^6^A37 nucleotide in the ASL would fit snugly in the active site groove of TcdA (Fig. 9C), in close proximity to active-site residues and ATP. Although very little is known about how TcdA cyclizes t^6^A37, it is safe to assume that the *N* ^6^-threonylcarbamoyl side chain of t^6^A37 will have to reach inside the ATP-binding pocket for the reaction to proceed, since ATP hydrolysis has been shown to occur concomitantly with reaction turnover. Further research will be necessary to elucidate the mechanistic details of the interaction, including the relative positions of the TcdA catalytic residues, the ATP substrate, and the t^6^A37 side chain.

Interestingly, the TcdA loop that is not defined in our crystal structures (residues 214–236) and that extends out of the K^+^ binding pocket right underneath the ATP binding site, would be well placed to interact with the tRNA molecules. It is currently unknown whether this loop will play a predominantly stabilizing, catalytic, or an intermediate, role. Since the loop sequence contains several positively charged residues and two potentially catalytic cysteine residues (Cys220 and Cys234), it could perform complex tasks both securing tRNA binding in a productive orientation and, perhaps, collaborating in the cyclization of t^6^A37. More extensive conformational changes at the TcdA-tRNA interface cannot be ruled out. For example, the largest crystallographic *B*-factors pertain to residues in helix α7 and the flap-like loop between α7-β7, facing toward the hypothetical tRNA interface. Those motifs could rearrange upon binding to a conformation where helix α6 and neighboring motifs could present shape and electrostatic properties resulting in an even more complementary binding surface for tRNA. A crystal structure of the TcdA-tRNA complex would shed light on these questions thereby allowing a more precise modeling of the catalytic mechanism for the TcdA-mediated biosynthesis of ct^6^A.

## Conclusions

**Fig 10:**
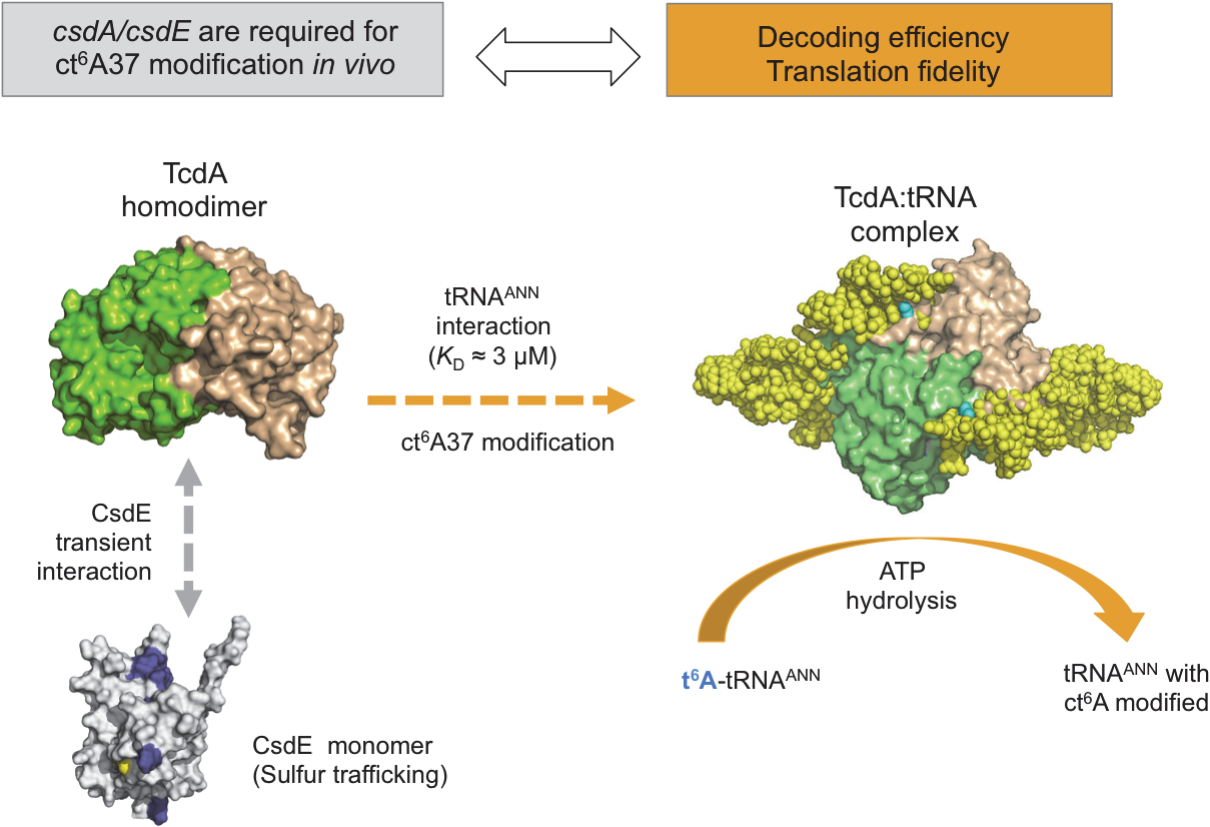
Interaction network for ct^6^A37-tRNA^ANN^ modification. TcdA (surface representation, monomer chains in green and wheat colors) interacts transiently but specifically with the sulfur acceptor CsdE (in grey, with TcdA-binding surface patches in blue and Cys61 in yellow), linking with the CsdA-CsdE cysteine desulfurase system and sulfur trafficking, which are known to be required for ct^6^A37 synthesis *in vivo* [16]. The CsdE-TcdA transient interaction is represented by a grey double-headed arrow. TcdA interacts with tRNA^ANN^ (*K*_D_ in the μM range) in a 2:2 complex that harbors ATP-dependent t^6^A37 dehydratase activity. The ct^6^A37 hypermodification is important for decoding efficiency and translation fidelity.

The crystal structures of MoeB/E1-like TcdA in complex with ATP and AMP and the SAXS-based bead models of the TcdA-tRNA^Lys^(UUU) reveal the basis for the association between TcdA and tRNA^ANN^. The TcdA-tRNA^Lys^(UUU) is a 2:2 complex in which two tRNA molecules bind independently to positively charged surfaces encompassing both TcdA chains. In addition, TcdA can interact transiently with the sulfur-acceptor CsdE through a speficic surface patch in the vicinity to the catalytic Cys61. These results provide a molecular basis for understanding the tRNA hypermodification function of TcdA that ensures decoding efficiency and translation fidelity of tRNA^ANN^ by the ribosome, and its connection with the sulfur trafficking systems that support TcdA function *in vivo* (Fig. 10).

## Supporting Information

S1 Fig Structure of t^6^A37 in the anticodon stem loop (ASL) of tRNA^Lys^(UUU). (PDF)

S2 Fig Guinier plots. (**a**) *E*. *coli* TcdA·ATP·Mg^2+^, (**b**) free tRNA^Lys^(UUU), (**c**) *E*. *coli* TcdA·ATP·Mg^2+^ + tRNA^Lys^(UUU), and (**d**) BMOE cross-linked *E*. *coli* TcdA-CsdE complex.
(PDF)

S3 Fig Cys66 is located in the active site of TcdA. (PDF)

S4 Fig Structural comparison of TcdA and MoeB. (PDF)

S5 Fig SAXS shape of BMOE-crosslinked CsdE-TcdA complex. (PDF)

S1 Table Summary of SAXS invariant parameters as inferred from data processing and analysis with ScÅtter, Primus, and GNOM. (PDF)

S2 Table Comparison of molecular mass estimates calculated from SAXS and SE-AUC. (PDF)

S1 Text Analysis of SAXS data for the BMOE cross-linked TcdA-CsdE complex. (PDF)
