# Correction: The Crystal Structure and Small-Angle X-Ray Analysis of CsdL/TcdA Reveal a New tRNA Binding Motif in the MoeB/E1 Superfamily

**DOI:** 10.1371/journal.pone.0134070

**Published:** 2015-07-24

**Authors:** Miguel López-Estepa, Ana Ardá, Martin Savko, Adam Round, William E. Shepard, Marta Bruix, Miquel Coll, Francisco J. Fernández, Jesús Jiménez-Barbero, M. Cristina Vega

There are multiple errors in Table 1. Please see the corrected Table 1 here.

**Table 1:** Crystallographic data processing and refinement statistics.

	TcdA-ATP	TcdA-AMP
PDB code	4D79	4D7A
**Data collection**		
Wavelength (Å)	0.9795	0.9801
Resolution range (Å)	41.13–1.77 (1.83–1.77)	41.14–1.80 (1.86–1.80)
Space group	*P* 1 21 1	*P* 1 21 1
Unit cell dimensions		
*a*, *b*, *c* (Å)	65.3, 96.7, 82.8	65.7, 97.2, 83.2
β (°), α = γ = 90°	90, 111.2, 90	90, 111.6, 90
Total reflections	312,887 (11,351)	351,361 (33,129)
Unique reflections	89,883 (5907)	87,219 (8261)
Multiplicity	3.5 (1.9)	4.0 (4.0)
Completeness (%)	95.96 (63.90)	97.14 (92.68)
Mean *I*/σ(*I*)	18.97 (3.75)	19.93 (4.05)
Wilson B-factor	23.95	21.59
R-merge	0.08388 (0.663)	0.04687 (0.412)
R-meas^a^	0.09797	0.05407
CC1/2^b^	0.995 (0.556)	0.999 (0.0.875)
CC*^c^	0.999 (0.846)	1.000 (0.966)
**Refinement**		
R-work	0.1416 (0.2994)	0.1396 (0.1839)
R-free	0.1833 (0.3236)	0.1768 (0.2350)
# non-H atoms	8167	8064
# Protein atoms	7484	7411
# Ligand atoms	172	46
# Water	511	607
Protein residues	996	974
RMS(bonds) (Å)	0.011	0.007
RMS(angles) (°)	1.430	1.01
Ramachandran analysis		
Favored/Allowed/Outlier (%)	98.0/2.0/0.0	98.0/2.0/0.0
Clashscore	1.88	1.93
Average *B*-factor (Å^2^)	34.40	32.10
Protein	33.60	31.50
Ligands	60.80	49.70
Solvent	38.00	37.20

^a^Rmeas = Σ_hkl_ (n/n-1)^1/2^ Σ_i_ |I_i_(hkl)-<I(hkl)>| / ΣΣ_i_ I_i_(hkl); where i is the ith measurement of reflection (hkl) and <I(hkl)> is the average over symmetry related observations of a unique reflection (hkl).

^b^CC1/2 is the Pearson correlation coefficient calculated between two random half data sets.

^c^CC* is the CC of the full data set against the true intensities, estimated from CC* = [2 CC1/2/(1+CC1/2)]^1/2^.

There is an error in the last sentence of the “TcdA crystallization, structure determination and refinement” subsection of the Materials and Methods. The correct sentence is: A data set for the AMP complex was collected to 1.80-Å resolution at the PROXIMA 2A beamline (Synchrotron SOLEIL, Paris, France). All the data sets were integrated with XDS [26] and scaled with Aimless [27] from the CCP4 suite of programs [28] (Table 1).

There are errors in the fifth and sixth sentences of the “Crystal structure of TcdA” subsection of the Results and Discussion. The correct sentences are: To shed light onto the structural basis for the tRNA binding and ct^6^A synthetic properties of TcdA-ATP, we determined the crystal structure of *E*. *coli* TcdA (Table 1 and Fig 2) loaded with ATP to 1.77 Å resolution (R/R_free_ values of 0.141/0.183) (Fig 3A) and AMP to 1.80 Å resolution (R/R_free_values of 0.139/0.176) (Fig 3B). The asymmetric unit contained four TcdA chains arranged in two independent dimers, with a solvent content of 39%.
