# Supplementary material for: The Crystal Structure and Small-Angle X-Ray Analysis of CsdL/TcdA Reveal a New tRNA Binding Motif in the MoeB/E1 Superfamily
Source: PLoS One. 2015 Apr 21;10(4):e0118606. doi: 10.1371/journal.pone.0118606 (PMC4405576; doi:10.1371/journal.pone.0118606)
Supplement: S1 Fig — (PDF) [file pone.0118606.s001.pdf]

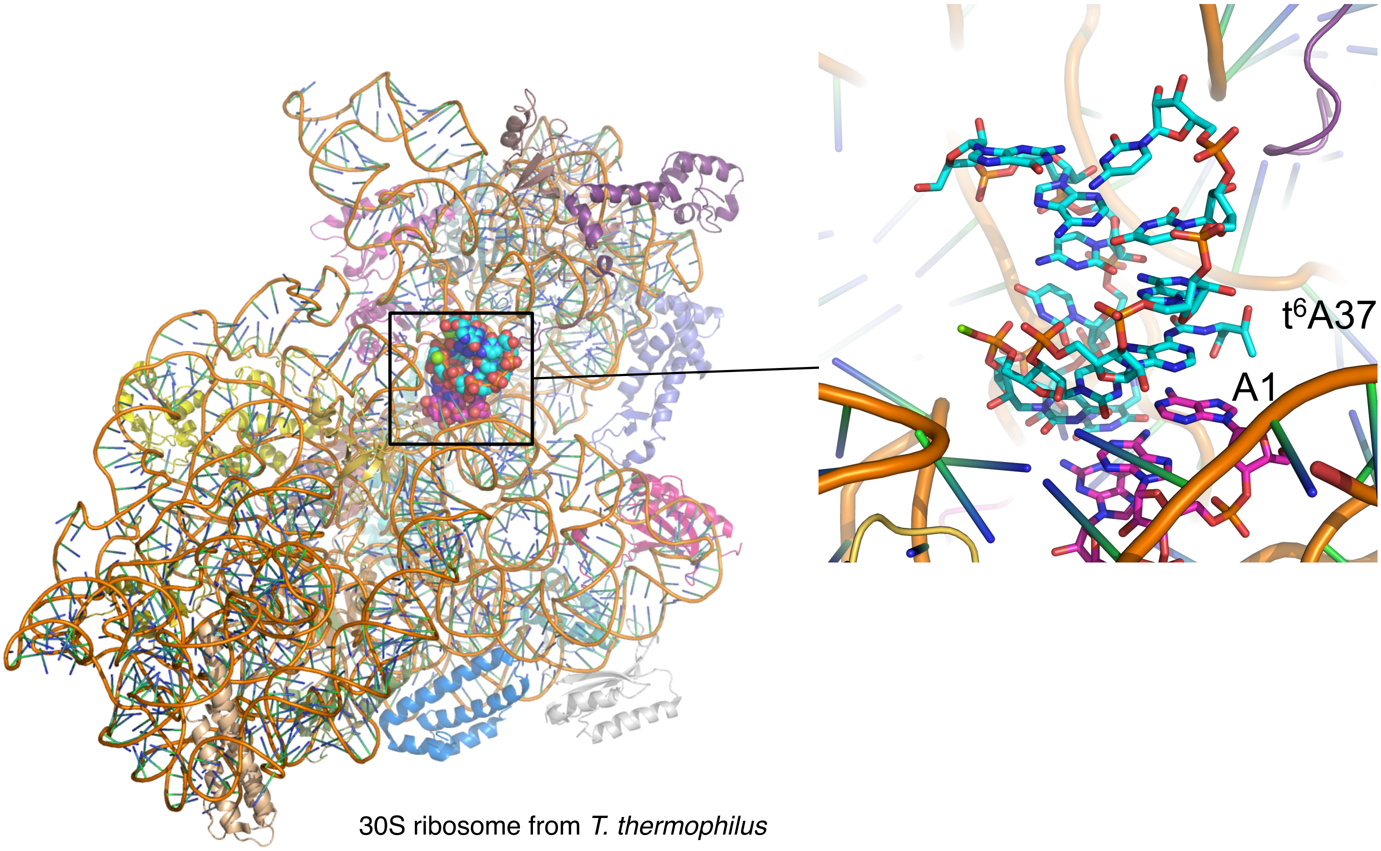

Figure S1. Structure of t^{6}A37 in the anticodon stem loop (ASL) of tRNA^{Lys}(UUU). As observed in complex with AAA-mRNA bound in the A-site of the decoding center of *Thermus thermophilus* 30S ribosome (PDB 1XMO) [1]. Inset: closeup of the modification-dependent ASL of tRNA^{Lys}(UUU) (C atoms in cyan) in complex with AAA-mRNA (carbon atoms in violet), with the positions of t^{6}A37 and A1 from the mRNA codon labeled.

1. Murphy FVt, Ramakrishnan V, Malkiewicz A, Agris PF (2004) The role of modifications in codon discrimination by tRNA(Lys)UUU. Nat Struct Mol Biol 11: 1186-1191.

Supplementary Information: The Crystal Structure and Small-Angle X-Ray Analysis of CsdL/TcdA reveal a new tRNA binding motif in the MoeB/E1 superfamily. M. López-Estepa, A. Ardá, M. Savko, A. Round, W.E. Shepard, M. Bruix, M. Coll, F.J. Fernández, J. Jiménez-Barbero, M.C. Vega. *PLoS ONE*, 2015.
