# Supplementary material for: The Crystal Structure and Small-Angle X-Ray Analysis of CsdL/TcdA Reveal a New tRNA Binding Motif in the MoeB/E1 Superfamily
Source: PLoS One. 2015 Apr 21;10(4):e0118606. doi: 10.1371/journal.pone.0118606 (PMC4405576; doi:10.1371/journal.pone.0118606)
Supplement: S2 Fig — (a) E. coli TcdA·ATP·Mg2+, (b) free tRNALys(UUU), (c) E. coli TcdA·ATP·Mg2+ + tRNALys(UUU), and (d) BMOE cross-linked E. coli TcdA-CsdE complex. (PDF) [file pone.0118606.s002.pdf]

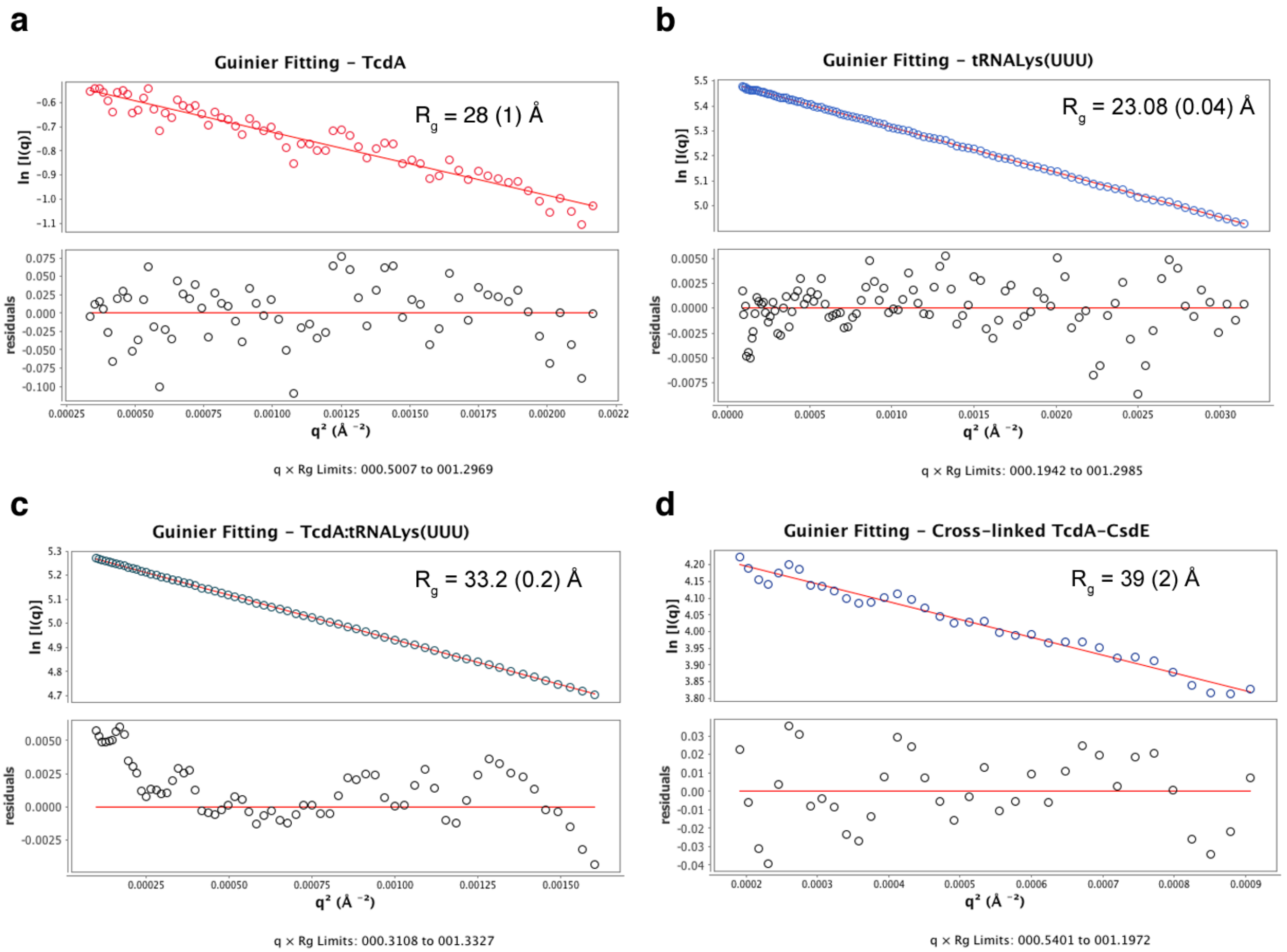

Figure S2. Guinier plots. (**a**) *E. coli* TcdA·ATP·Mg^{2+}, (**b**) free $tRNA^{Lys}$(UUU), (**c**) *E. coli* TcdA·ATP·Mg^{2+} + $tRNA^{Lys}$(UUU), and (**d**) BMOE cross-linked *E. coli* TcdA-CsdE complex.
