# Supplementary material for: The Crystal Structure and Small-Angle X-Ray Analysis of CsdL/TcdA Reveal a New tRNA Binding Motif in the MoeB/E1 Superfamily
Source: PLoS One. 2015 Apr 21;10(4):e0118606. doi: 10.1371/journal.pone.0118606 (PMC4405576; doi:10.1371/journal.pone.0118606)
Supplement: S3 Fig — (PDF) [file pone.0118606.s003.pdf]

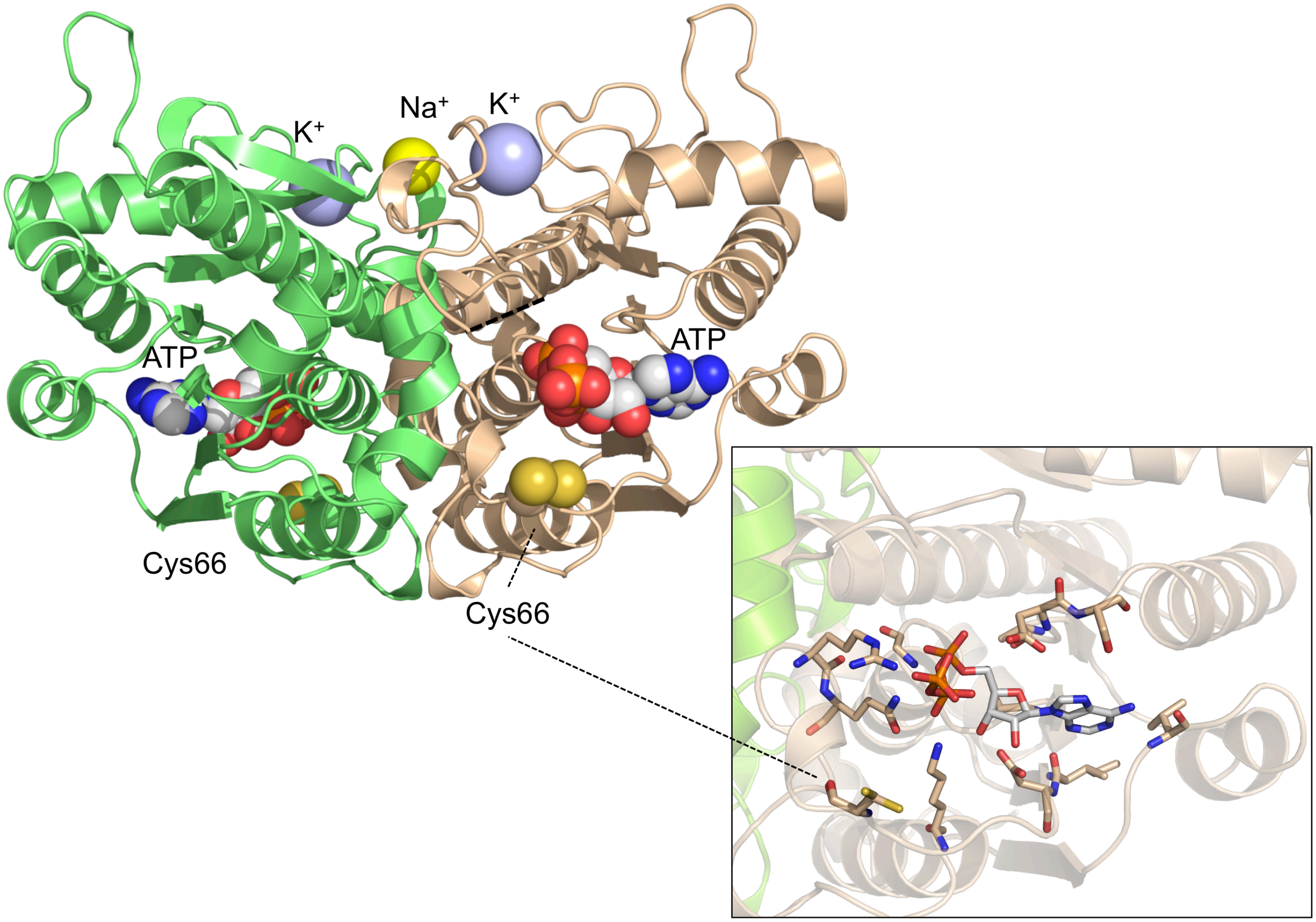


**Figure S3. Cys66 is located in the active site of TcdA.** Ribbon structure of the TcdA dimer (in chain colors), with ATP, Cys66, $K^+$ and $Na^+$ in spheres (CPK colors). Cys66 is located in the active site of TcdA, defined by its proximity to ATP, although there are no direct interactions between Cys66 atoms and ATP. The inset shows the active site pocket with ATP, ATP-contacting residues (as in **Figure 2a** in the manuscript) and Cys66 represented in sticks. Cys66 side chain is split in two alternate conformations which are nearly equally occupied, as shown in the overall dimer view and the inset view. The disordered loop (residues 214-236) is shown as a dashed line; two additional cysteine residues, Cys220 and Cys234, fall into the disordered loop and, therefore, cannot be located in the structure.
