# Supplementary material for: The Crystal Structure and Small-Angle X-Ray Analysis of CsdL/TcdA Reveal a New tRNA Binding Motif in the MoeB/E1 Superfamily
Source: PLoS One. 2015 Apr 21;10(4):e0118606. doi: 10.1371/journal.pone.0118606 (PMC4405576; doi:10.1371/journal.pone.0118606)
Supplement: S4 Fig — (PDF) [file pone.0118606.s004.pdf]

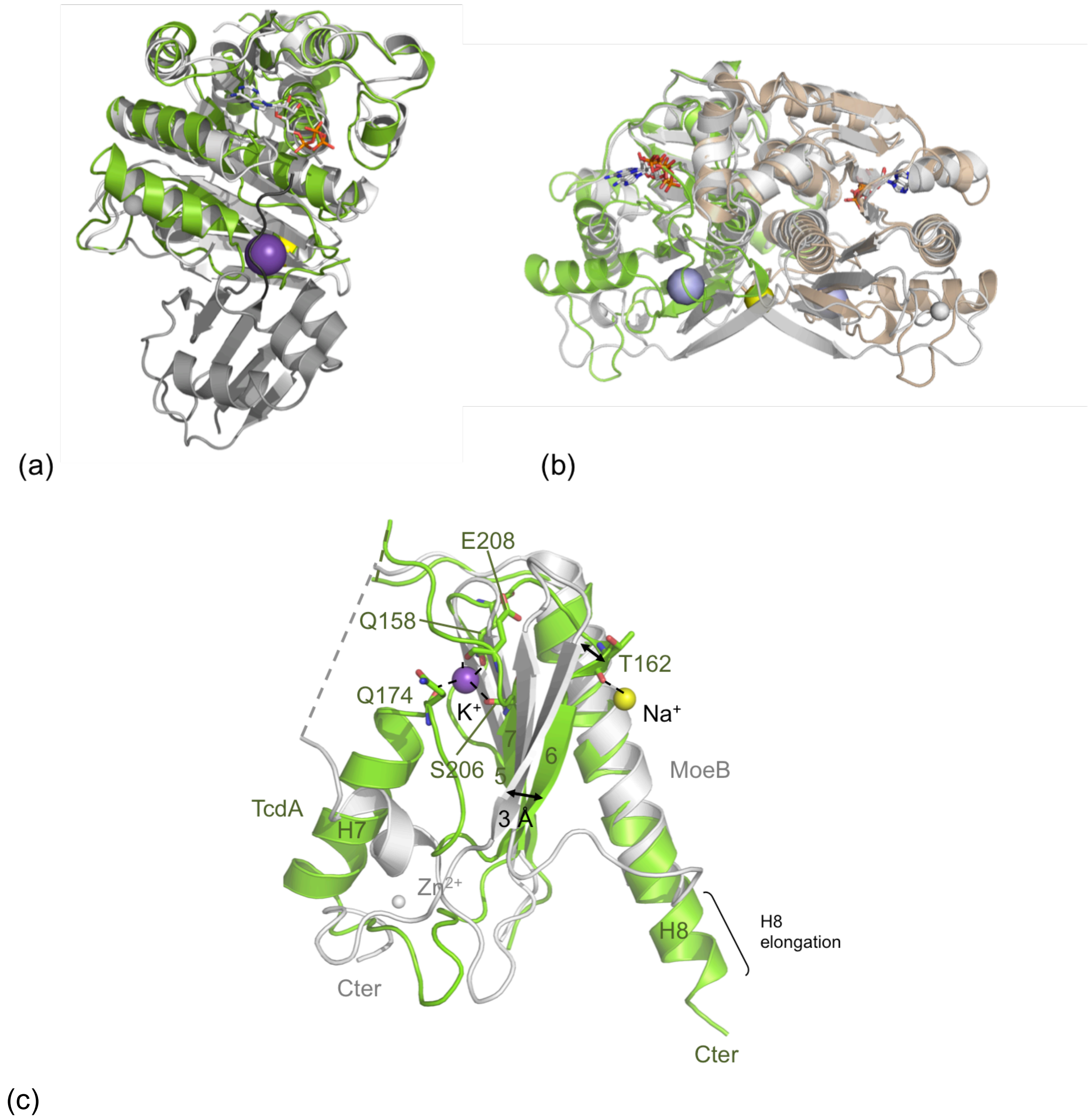

Supplementary Information: The Crystal Structure and Small-Angle X-Ray Analysis of CsdL/TcdA reveal a new tRNA binding motif in the MoeB/E1 superfamily. M. López-Estepa, A. Ardá, M. Savko, A. Round, W.E. Shepard, M. Bruix, M. Coll, F.J. Fernández, J. Jiménez-Barbero, M.C. Vega. *PLoS ONE*, 2015.

Figure S4. Structural comparison of TcdA and MoeB. (**a**) Superposition of a TcdA monomer (green) and MoeB-MoeD (light and dark shades of grey; PDB 1JWA [1]). (**b**) Superposition of a TcdA dimer (chains in green and grey) with MoeB (brown; PDB 1JWA [1]), highlighting the similar E1-like fold as well as the differences in fold topology and metal binding sites. K^+ and Zn^{2+} are shown as a purple or gray spheres, respectively. (**c**) Detail of the superposition of the divergent C-terminal domain of TcdA with that of MoeB, highlighting the major structural differences. In particular, differences in the strands β5, β6 and β7 are responsible for the organization of two new metal-binding sites absent in all known E1-like activating enzymes, a K^+ ion binding per TcdA chain and an interfacial Na^+ pocket. Missing residues in mobile loops are indicated by dashed lines.

1. Lake MW, Wuebbens MM, Rajagopalan KV, Schindelin H (2001) Mechanism of ubiquitin activation revealed by the structure of a bacterial MoeB-MoaD complex. Nature 414: 325-329.
