# Supplementary material for: The Crystal Structure and Small-Angle X-Ray Analysis of CsdL/TcdA Reveal a New tRNA Binding Motif in the MoeB/E1 Superfamily
Source: PLoS One. 2015 Apr 21;10(4):e0118606. doi: 10.1371/journal.pone.0118606 (PMC4405576; doi:10.1371/journal.pone.0118606)
Supplement: S5 Fig — (PDF) [file pone.0118606.s005.pdf]

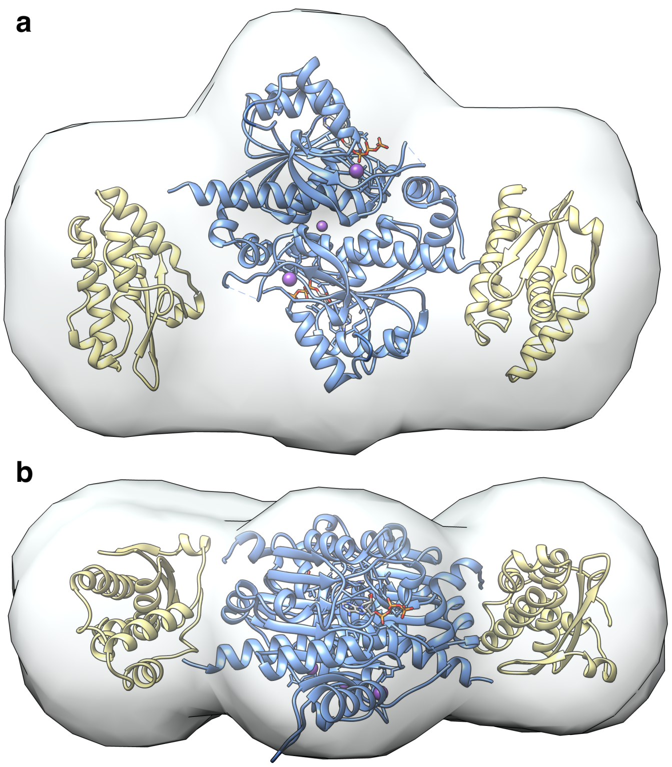

Figure S5. SAXS shape of BMOE-crosslinked CsdE-TcdA complex. Representative DAMMIF shape (semitransparent white surface) reconstructed imposing *P*2 symmetry with TcdA (blue) and CsdE (yellow) models fitted inside. Two orientations related by a 90º rotation around a horizontal axis are shown (**a** and **b**).

Supplementary Information: The Crystal Structure and Small-Angle X-Ray Analysis of CsdL/TcdA reveal a new tRNA binding motif in the MoeB/E1 superfamily. M. López-Estepa, A. Ardá, M. Savko, A. Round, W.E. Shepard, M. Bruix, M. Coll, F.J. Fernández, J. Jiménez-Barbero, M.C. Vega. *PLoS ONE*, 2015.
