# Supplementary material for: The Crystal Structure and Small-Angle X-Ray Analysis of CsdL/TcdA Reveal a New tRNA Binding Motif in the MoeB/E1 Superfamily
Source: PLoS One. 2015 Apr 21;10(4):e0118606. doi: 10.1371/journal.pone.0118606 (PMC4405576; doi:10.1371/journal.pone.0118606)
Supplement: S1 Table — (PDF) [file pone.0118606.s006.pdf]

Table S1. Summary of SAXS invariant parameters as inferred from data processing and analysis with ScÅtter, Primus, and GNOM.

	**TcdA**	**tRNA**	**TcdA-tRNA**	**TcdA-CsdE**
I(0) (Guinier)	0.627 ± 0.006	242.1 ± 0.1	200.0 ± 0.2	73.6 ± 0.6
R_g (Guinier)	28 ± 1 Å	23.08 ± 0.04 Å	33.2 ± 0.1 Å	39 ± 2 Å
R_g (Real space)	25 ± 1 Å	23.8 ± 0.1 Å	32.6 ± 0.6 Å	36.4 ± 0.5 Å
V_C (Guinier)	211.89 Å^3	243.1 Å^3	606.5 Å^3	231.2 Å^3
Volume (Porod)	132.63 Å^3	40,225 Å^3	297,610 Å^3	256,265 Å^3
Protein mass (Guinier)	62,333 Da	n.a.	90,035 Da	154,919 Da
RNA mass (Guinier)	n.a.	24,221 Da	80,919 Da	n.a.
Average R	33.04 Å	30.27 Å	42.8 Å	47.57 Å
D_{max}	72 Å	75 Å	109 Å	108 Å
R_C	16.9 ± 1 Å	14.41 ± 0.09 Å	23.6 ± 0.7 Å	21.5 ± 0.5 Å
P_X	3.1 ± 0.1	3.90 ± 0.03	2.7 ± 0.02	2.30 ± 0.02

Supplementary Information: The Crystal Structure and Small-Angle X-Ray Analysis of CsdL/TcdA reveal a new tRNA binding motif in the MoeB/E1 superfamily. M. López-Estepa, A. Ardá, M. Savko, A. Round, W.E. Shepard, M. Bruix, M. Coll, F.J. Fernández, J. Jiménez-Barbero, M.C. Vega. *PLoS ONE*, 2015.
