# Supplementary material for: The Crystal Structure and Small-Angle X-Ray Analysis of CsdL/TcdA Reveal a New tRNA Binding Motif in the MoeB/E1 Superfamily
Source: PLoS One. 2015 Apr 21;10(4):e0118606. doi: 10.1371/journal.pone.0118606 (PMC4405576; doi:10.1371/journal.pone.0118606)
Supplement: S2 Table — (PDF) [file pone.0118606.s007.pdf]

Table S2. Comparison of molecular mass estimates calculated from SAXS and SE-AUC.

	**Theoretical mass[1] (Da)**	**SAXS (Guinier)[2]**	**SAXS (Porod)[3]**	**SE-AUC[4]**
TcdA monomer	28,430.8	n.a.	n.a.	n.a.
TcdA dimer	57,969.2	62,333.0	66,315.0	63,531.0
$tRNA^{Lys}$(UUU)	23,440.3	24,221.0	20,112.5	26,066.0
TcdA-tRNA	104,849.5	90,035.0[5]	148,805.0	114,297.0
CsdE	16,760.1	n.a.	n.a.	n.a.
TcdA-CsdE	91,929.5	154,919.0	128,132.0	n.a.

[1] Theoretical masses were calculated from the primary sequence minus the initiation methionine, which is mostly absent in recombinant TcdA and CsdE (data not shown). The masses of two ATP^{4-} (2 × 503.18 Da), two K^+ (2 × 39.1 Da) and one Na^+ (23.0 Da) were added to that of the TcdA dimer, TcdA-tRNA and TcdA-CsdE cross-linked complex. The thoretical mass for $tRNA^{Lys}$(UUU) does not take into account the mass change due to the tRNA chemical modifications and accordingly should be taken as an approximation. For the cross-linked TcdA-CsdE complex, the net mass added by the cross-linking reagent (BMOE, 220.05 Da) was added once per CsdE molecule in the cross-linked complex.

[2] Molecular mass estimate derived from Guinier approximation.

[3] Molecular mass estimate derived from the Porod volume.

[4] Molecular mass estimate calculated from SE-AUC data (see **Material and Methods** in the publication).

[5] Molecular mass estimate assumes protein only.
