# Supplementary material for: The Crystal Structure and Small-Angle X-Ray Analysis of CsdL/TcdA Reveal a New tRNA Binding Motif in the MoeB/E1 Superfamily
Source: PLoS One. 2015 Apr 21;10(4):e0118606. doi: 10.1371/journal.pone.0118606 (PMC4405576; doi:10.1371/journal.pone.0118606)
Supplement: S1 Text — (PDF) [file pone.0118606.s008.pdf]

Text S1. Analysis of SAXS data for the BMOE cross-linked TcdA-CsdE complex

Preparative amounts of the BMOE cross-linked TcdA-CsdE complex were prepared by using the methods outlined in the manuscript. In brief, TcdA and CsdE were mixed at approximately equimolar amounts in 20 mM sodium/potassium phosphate, 450 mM NaCl, 0.5 mM EDTA, pH 7.4, and treated with 1 mM TCEP to remove unwanted disulfide bridges (30 min at 25 ºC). Then, the cross-linking reagent BMOE [bis(maleimido)ethane, Pierce cat. no. 22322] was added to 0.2 mM final concentration and the reaction incubated for 1 h at 25 ºC. The net mass added by a BMOE cross-link is 220.05 Da. Since BMOE has a short spacer arm (7-atom, 8-Å long), it is capable of cross-linking sulfhydryl groups that are in close proximity across protein-protein interfaces, which, therefore, may represent specific complexes with short half-time lives (i.e., transient protein complexes). Complete conversion of free TcdA into cross-linked complex was verified by SDS-PAGE, and the cross-linked complex was isolated by nickel-affinity chromatography (exploiting the C-terminal hexahistidine tag present in CsdE) and gel filtration (to eliminate excess free CsdE). The purified complex was submitted for SAXS data collection using the batch setup at the BM29 BioSAXS beam line at the ESRF, Grenoble (France).

SAXS data was processed using standard methods and model-independent parameters or SAXS invariants were calculated (see the manuscript for details) (**Table S1 and Figure S5**). Since there were signs of concentration-dependent aggregation in the sample at very low angles, we cropped the first data points to alleviate potentially adverse effects during the subsequent analyses. Analysis of the Kratky plots in ScÅtter revealed a significant degree of flexibility in the cross-linked complex, even though it behaved like a folded, compact particle (Porod's exponent, P_X = 2.3). This behavior is consistent with cross-linked protein molecules that are well folded and tethered by short linkers, in this case, 8-Å BMOE cross-links.

Attempts to reconstruct the molecular envelope of cross-linked TcdA-CsdE by *ab initio* approaches (DAMMIF) generated congruent shapes. Out of 40 *ab initio* models calculated with DAMMIF without imposing symmetry, only 3 were discarded and the remaining 37 models had an average NSD = 0.630 ± 0.084. Applying *P*2 symmetry to shape restoration generated models with a greater variability (average NSD = 1.206 ± 0.391, 40 independent models) but which improve the visual quality of the model fit to the *ab initio* models, indicating that the cross-linked CsdE subunits might roughly occupy pseudo-symmetrical positions across the TcdA dimer, even if multiple equivalent positions and orientations could be possible within the complex. A representative model with fitted TcdA (blue) and CsdE (yellow) models is depicted in **Figure S5**.
